# Mapping Composition Evolution through Synthesis, Purification, and Depolymerization of Random Heteropolymers

**DOI:** 10.1021/jacs.3c13909

**Published:** 2024-02-22

**Authors:** Hao Yu, Luofu Liu, Ruilin Yin, Ivan Jayapurna, Rui Wang, Ting Xu

**Affiliations:** † California Institute for Quantitative Biosciences, University of California, Berkeley, Berkeley, California 94720, United States; ‡ Department of Chemical and Biomolecular Engineering, University of California, Berkeley, Berkeley, California 94720, United States; § Department of Chemistry, University of California, Berkeley, Berkeley, California 94720, United States; ∥ Department of Materials Science and Engineering, University of California, Berkeley, Berkeley, California 94720, United States; ⊥ Departent of Materials Science and Engineering, University of California, Berkeley, Berkeley, California 94720, United States

## Abstract

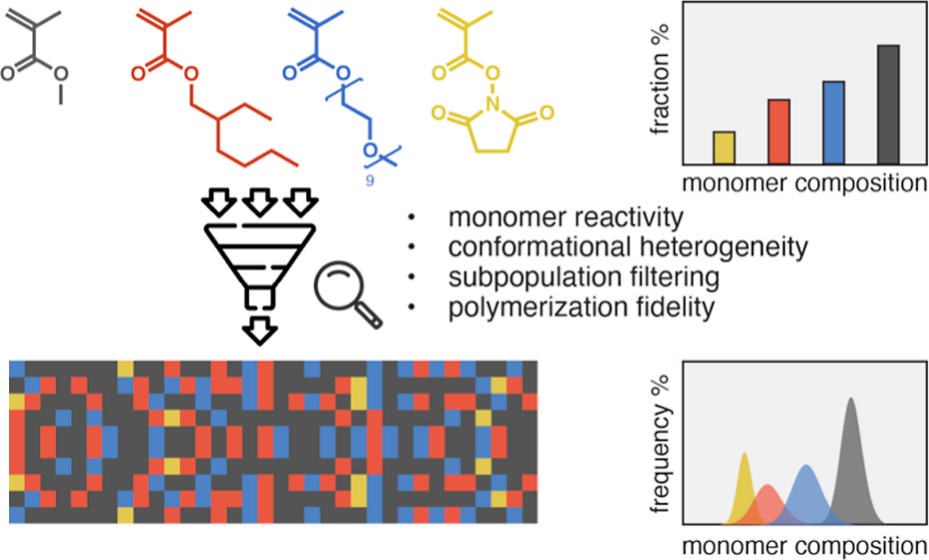

Random heteropolymers (RHPs) consisting of three or more comonomers have been routinely used to synthesize functional materials. While increasing the monomer variety diversifies the side-chain chemistry, this substantially expands the sequence space and leads to ensemble-level sequence heterogeneity. Most studies have relied on monomer composition and simulated sequences to design RHPs, but the questions remain unanswered regarding heterogeneities within each RHP ensemble and how closely these simulated sequences reflect the experimental outcomes. Here, we quantitatively mapped out the evolution of monomer compositions in four-monomer-based RHPs throughout a design-synthesis-purification-depolymerization process. By adopting a Jaacks method, we first determined 12 reactivity ratios directly from quaternary methacrylate RAFT copolymerization experiments to account for the influences of competitive monomer addition and the reversible activation/deactivation equilibria. The reliability of in silico analysis was affirmed by a quantitative agreement (<4% difference) between the simulated RHP compositions and the experimental results. Furthermore, we mapped out the conformation distribution within each ensemble in different solvents as a function of monomer chemistry, composition, and segmental characteristics via high-throughput computation based on self-consistent field theory (SCFT). These comprehensive studies confirmed monomer composition as a viable design parameter to engineer RHP-based functional materials as long as the reactivity ratios are accurately determined and the livingness of RHP synthesis is ensured.

## Introduction

Heteropolymers synthesized by copolymerizing three or more monomers have been extensively used in plastic industries, including lubrication, film packaging, injection molding, and surface modification.^1−5^ Their recent applications extend to catalysis,^6−10^ drug delivery,^11−13^ and protein mimics.^14−20^ Advances in reversible-deactivation radical polymerization (RDRP) have enabled the synthesis of heteropolymers with good repeatability,^21^ and most heteropolymer designs have focused on optimizing monomer composition.^18,22,23^ However, the inherent stochastic nature of sequence control in multimonomer copolymerization leads to many questions regarding the heterogeneities within an ensemble in composition, as well as in segmental and monomeric sequences.^11^ Despite the limited successes in synthesizing and sequencing synthetic polymers with monomeric sequence specificity, we must also answer how the synthesis and purification process may affect the heterogeneities of sequence and property in a statistical polymer chain ensemble and identify key design parameters.^24,25^

In multimonomer copolymerization, the intrinsic reactivity of each monomer and local composition determine the arrangement of monomers along a heteropolymer chain. Understanding the intrinsic reactivity among comonomers allows for tuning segmental sequences and biasing the flanking sequences of particular residues. However, determining the reactivity ratios of comonomers becomes challenging when the number of monomers exceeds three, due to the complex, interdependent reaction kinetics and the mathematical models involved.^26−28^ Therefore, reactivity ratios obtained from binary copolymerization pairs have been commonly used,^29−31^ although neglecting the competitive monomer addition in multimonomer systems can lead to substantial deviations between experimental results and theoretical predictions.^32,33^ RDRP techniques have greatly facilitated the synthesis of heteropolymers.^21^ Yet, the reversible activation/deactivation equilibria in RDRP can also influence the chain propagation kinetics,^34^ thereby distorting the predictions from in silico designs.^35^ These factors need to be taken into account for an accurate determination of reactivity ratios, which are essential inputs for heteropolymer design and analysis.^26,36^

Heteropolymers are polydisperse in chain length, molecular weight, monomer composition, and sequence. The intrinsic sequence heterogeneity of heteropolymers raises questions about the possible elimination of heteropolymer subpopulations during post-polymerization purifications. Bridging these gaps requires systematic investigations of the entire heteropolymer preparation process. Early works from Shea attempted an affinity chromatography approach to separate chains from a pool of heteropolymers.^37^ They demonstrated that a subpopulation of the as-synthesized heteropolymers can exhibit more than 1000-fold higher affinity for a target protein than the average affinity.^38^ Yet, there have been limited methods available for separating subpopulations within a heteropolymer ensemble.^39−42^ Prior work has demonstrated that controlling the distributions of segmental monomer blocks in heteropolymers at the single-chain level can achieve protein-like properties.^20^ This notion is supported by recent computational studies which have revealed that the morphology of single-chain nanoparticles (SCNPs) can be tuned by generalizable sequence patterning characteristics such as consecutive monomer segment length.^43,44^ Thus, there is a clear need to develop new approaches to assess the sequence and property heterogeneities within heteropolymer ensembles.

**Scheme 1:**
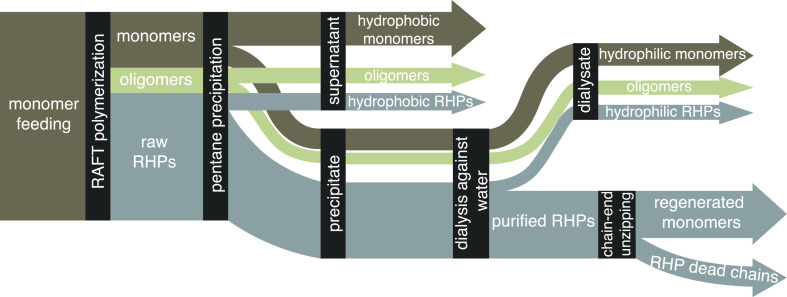
Composition Evolution of RHPs over the Course of the Entire Preparation Process Due to composition drift and chain removal during purification, the composition of the final RHP products may differ from the initial feeding composition.

Average polymer composition has long served as a key design parameter for tailoring the properties of heteropolymer materials.^45,46^ Fine-tuning chain behaviors in statistical heteropolymer ensembles demands the consideration of sequence patterning, such as consecutive monomer blocks.^47^ This aspect is also directly tied to the composition through the intrinsic reactivity of individual monomers. Therefore, we tracked the monomer composition evolution throughout the entire preparation process and investigated how the synthesis and purification manage the heterogeneities inherent in random heteropolymer (RHP) ensembles. Specifically, we quantitatively mapped out the evolutionary trajectory of monomer compositions across three tiers: (i) raw RHP products; (ii) purified RHPs; and (iii) the living chain subpopulations [chain terminated by a chain transfer agent (CTA)] (Scheme 1). The results reveal negligible removal of RHP chains during antisolvent precipitation and dialysis, with no discernible impact on ensemble-level heterogeneity. To establish a robust framework for simulating RHP sequences, we determined the reactivity ratios for a set of four methacrylate monomers in quaternary RAFT copolymerization using a Jaacks method. Using the sequences simulated from a Monte Carlo algorithm, we analyzed 4000 unique coarse-grained RHP chains in a high-throughput manner. The results suggested that RHP’s monomer composition can be designed to induce a selective shifting and narrowing of conformation distribution at the ensemble level.

## Results and Discussion

### Determining Monomer Reactivity Ratios in Quaternary RAFT Copolymerization

**Figure 1:**
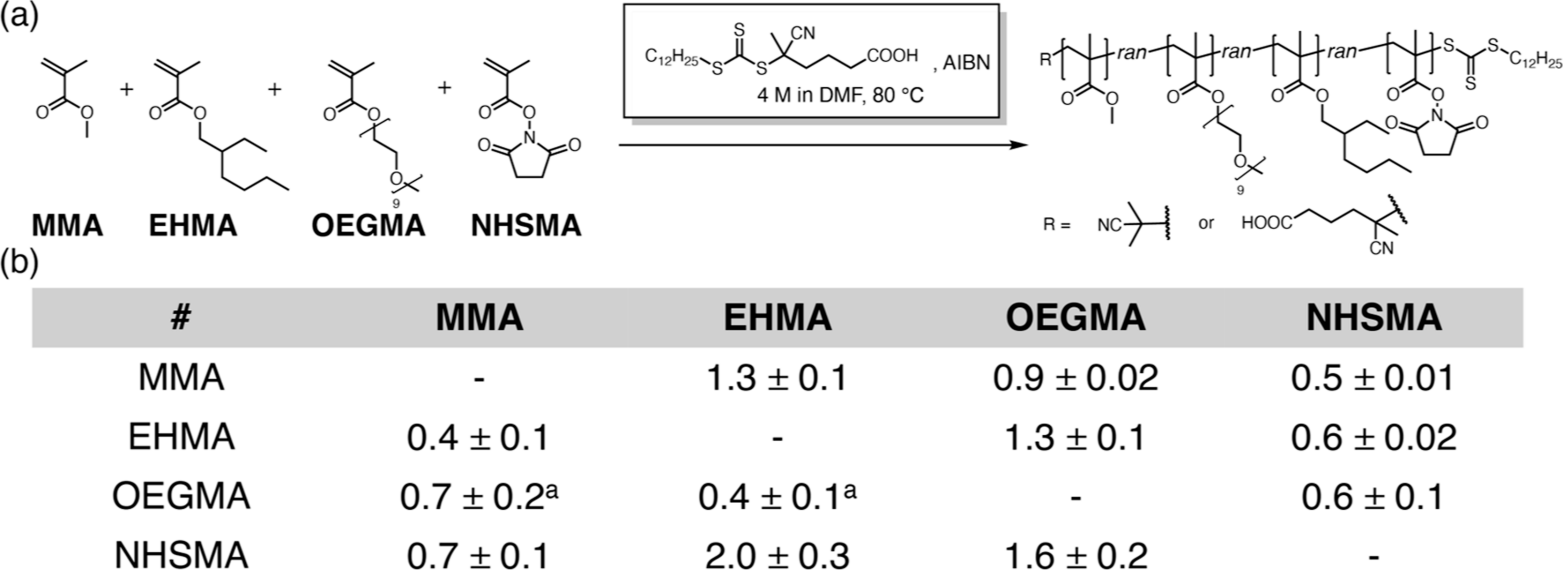
(a) Synthesis scheme of four-monomer RHPs. (b) Reactivity ratios determined in this work (*r*_row/Column_ = *k*_rowrow_/*k*_rowcolumn_). ^a^*r*_OEGMA/MMA_ and *r*_OEGMA/EHMA_ were determined separately by two-monomer copolymerization experiments to minimize analysis error due to peak overlap in the ^1^H NMR spectra.

We selected four monomers: methyl methacrylate (MMA), 2-ethylhexyl methacrylate (EHMA), oligo(ethylene glycol) methyl ether methacrylate (OEGMA; average *M*_n_ ∼ 500 Da), and methacrylic acid *N*-hydroxysuccinimide ester (NHSMA) (Figure 1a). MMA, EHMA, and OEGMA were routinely used to mimic amino acids in RHP synthesis.^18,48^ 3-Sulfopropyl methacrylate potassium salt (SPMA) was previously used as the fourth monomer. However, the chemical shifts of SPMA and EHMA largely overlap in the NMR spectra. We therefore chose NHSMA as the fourth monomer to facilitate subsequent characterizations. NHSMA has a pendent imide group and may exhibit different reactivity compared with the other three monomers. This can potentially boost composition drift during copolymerization, presenting supplementary measures to tune the monomer distribution in RHPs.

To account for the competitive monomer addition in multimonomer copolymerization, we used the Jaacks method for the four-monomer RHP copolymerization and determined all 12 reactivity ratios (Figure 1b, and Section S5).^49,50^ This method applies integrated Mayo–Lewis equations to multimonomer copolymerization systems. It allows for the simultaneous determination of multiple reactivity ratios in a single quaternary copolymerization experiment while accounting for the influence of competitive monomer addition. This approach avoids oversimplifying the kinetics of multimonomeric copolymerization by relying solely on reactivity ratios derived from binary copolymerization experiments.^32^ Following the Jaacks method, we performed the four-monomer RAFT polymerization with one monomer in large excess (molar ratio: 20:1:1:1) in each experiment (experiment details in Section S5). To account for the influence of RAFT equilibrium on the propagation kinetics, we conducted these experiments under the same conditions as those commonly used in RHP RAFT polymerization {using 4-cyano-4-[(dodecylsulfanylthiocarbonyl)sulfanyl]pentanoic acid as CTA and azobis(isobutyronitrile) (AIBN) as initiator at 80 °C in DMF}.

The four selected monomers exhibit distinct reactivity ratios, with values ranging between 0.4 and 2.0. In the four-monomer RAFT copolymerization, cross-propagation of the growing chain end toward MMA is more favorable than self-propagation (*r*_non-MMA monomer/MMA_ < 1), whereas self-propagation is generally preferred over cross-propagation toward OEGMA (*r*_EHMA/OEGMA_ and *r*_NHSMA/OEGMA_ > 1). These results suggest the important influence of the side-chain steric effects, with MMA having the smallest side group and OEGMA featuring a bulky side-chain. Notably, the NHSMA can lead to more significant composition drifts. Polymer chains with terminal NHSMA group undergo self-propagation approximately two times faster than cross-propagation toward EHMA (*r*_NHSMA/EHMA_ = 2.0), but 30% slower toward MMA (*r*_NHSMA/MMA_ = 0.7). Therefore, in RHP copolymerization, a higher prevalence of the NHSMA-MMA segment can be generated compared to the NHSMA-EHMA or NHSMA–NHSMA segments. Prior work has demonstrated that leveraging intrinsic monomer reactivity offers a facile method for governing segmental sequence.^51,52^ Additionally, employing a sequential addition approach can afford a more precise level of control over monomer distribution.^53,54^ We envision the investigation on monomer reactivity ratios here lays a foundation for effectively regulating comonomer distribution in RHPs, with adaptable applicability to various multimonomer systems.

### RHP Design and Sequence Simulation

**Figure 2:**
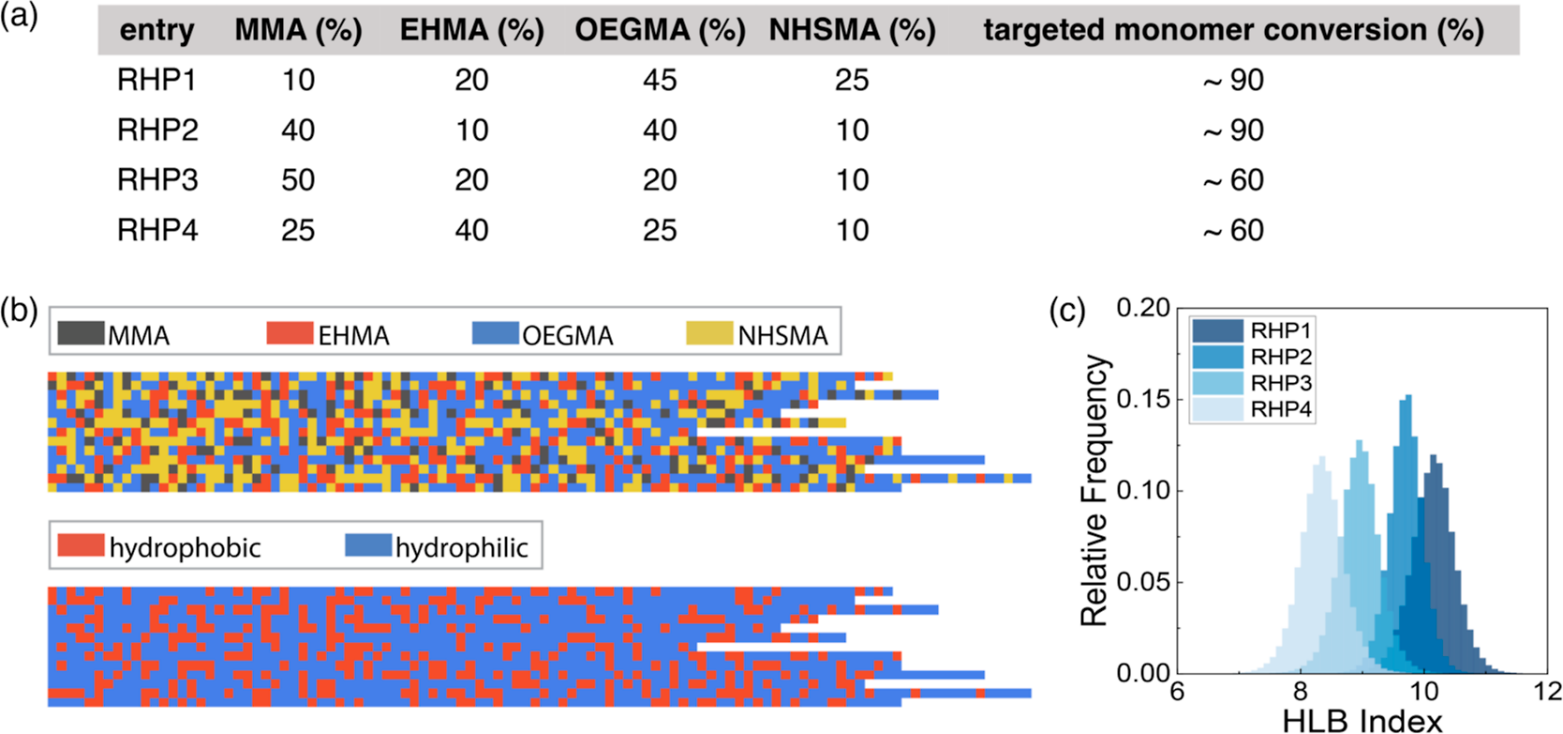
(a) Design of RHPs with increased hydrophobicity by increasing the compositions of hydrophobic monomers (MMA and EHMA) from **RHP1** to **RHP4**. (b) Random sequences sampled from simulated **RHP1** using measured reactivity ratios and targeted monomer conversion. The RHP sequences shown at the bottom were binarized into hydrophobic (MMA and EHMA) and hydrophilic (OEGMA and NHSMA) units to display the distributions of hydrophobic/hydrophilic segments. (c) Distribution of chain hydrophobicity in in silico-designed **RHP1–4**. Chain hydrophobicity was estimated using previously reported HLB values (MMA: 8.45, EHMA: 5.125, OEGMA: 11.42, and NHSMA: 12.775).^20,48^

Using the experimentally determined monomer reactivity ratios, we designed four model RHPs with the ratio of hydrophobic monomers (MMA and EHMA) increasing progressively from **RHP1** to **RHP4** (Figure 2a). This formulation leads to a progressive increase in the average chain hydrophobicity from **RHP1** to **RHP4**. We designed the monomer compositions based on previously reported RHPs consisting of MMA, EHMA, OEGMA, and SPMA,^18^ along with our empirical experience on finding an optimal balance in solubility. For example, maintaining a ratio of EHMA or NHSMA below 50% is needed to avoid polymer precipitation in DMF before reaching the desired monomer conversion (>60%) during polymerization. In contrast, a ratio of OEGMA above 10% is necessary to ensure the water solubility of the final products. In radical polymerization, there is a trade-off between achieving higher monomer conversion and ensuring control over the reaction. This is due to monomer depletion and potential loss of end-group fidelity at higher conversions. However, attaining high monomer conversion reduces purification cost and minimizes waste generation. Additionally, the extent of compositional drift during polymerization is contingent on overall monomer conversion.^55^ Therefore, we targeted **RHP1–4** for different overall monomer conversions as part of our studies into the optimal production strategies for RHP materials.

To facilitate our design, we simulated RHP sequences using a stochastic simulator based on Mayo–Lewis model, the *RHPapp*.^48,56^ This program simulates heteropolymer monomer sequences using a terminal model, assuming that the chain propagating kinetics is only contingent on the terminal monomer.^11,55^ Experimental inputs including feeding composition, global monomer conversion, and reactivity ratios were used to simulate RHP sequences by using a Monte Carlo algorithm (Section S1). A total of 100,000 RHP sequences were generated for each model RHP batch for statistical analysis. The four designed RHPs exhibit sequence heterogeneity within local segments and between different chains (Figures 2b and S34–S36). There is no discernible monomer gradient observed along the RHP chains.

To evaluate the RHP design, we estimated the average chain hydrophilicity using hydrophilic–lipophilic balance (HLB) values.^57^ The lower HLB values indicate greater hydrophobicity, whereas the higher values reflect increased hydrophilicity. The results indicate a consistent decrease in hydrophilicity from **RHP1** to **RHP4**, aligning with our intended design (Figure 2c). Collectively, this set of four designed model RHPs spans the representative range of hydrophobicity that is commonly encountered in the RHP design, benchmarked with poly(MMA) (HLB value: 8.45) and poly(OEGMA) (HLB value: 11.42). Across the entire range of HLB values, each of the four model polymers exhibits a well-differentiated average hydrophobicity (Figure 2c). We anticipate that these four model polymers will serve as benchmarks, improving the prediction of the solution behavior in heteropolymers for future designs.

**Figure 3:**
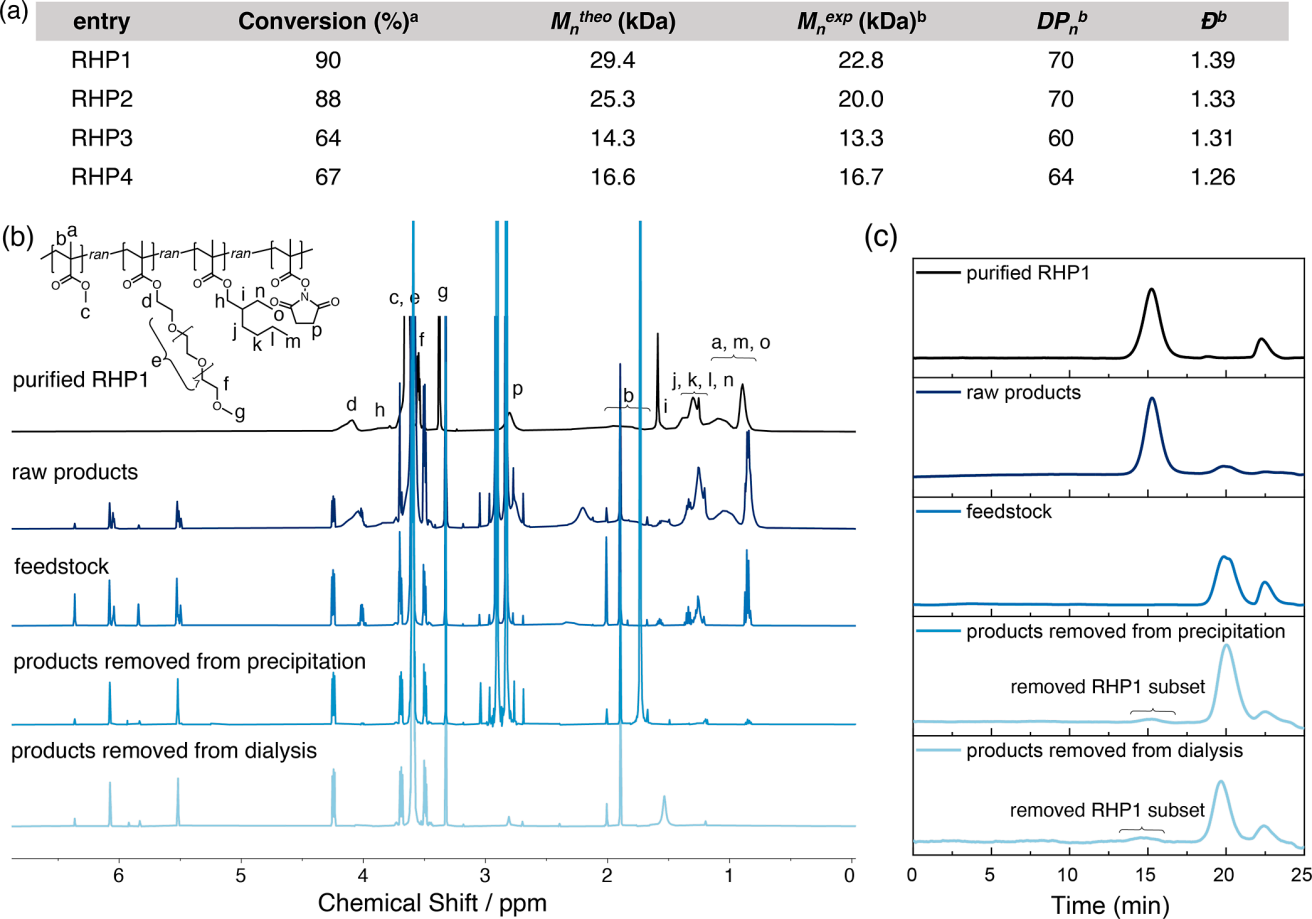
(a) Summary of the characterization results (averaged from three independent synthesis batches) of **RHP1–4**. ^a^Overall monomer conversion determined from pre- and post-polymerization aliquots ^b^determined using THF GPC with PMMA standards. DP_*n*_ was calculated using an averaged molecular weight based on the anticipated monomer composition. (b) Representative ^1^H NMR spectra (CDCl_3_) and (c) GPC traces (THF) of **RHP1** and intermediates during synthesis. Residual GPC solvent peak (THF) is shown at 22.5 min.

Recent studies on polymer diffusion kinetics during dialysis have revealed crucial influence of chemical composition on polymer retention.^58,59^ Given the inherent sequence heterogeneity of RHPs, it naturally leads to the question of whether certain RHP chains are removed during the purification process (Scheme 1). Thus, we monitored the composition evolution of **RHP1–4** at every stage of the preparation process (Figure 3a, synthesis details in Section S2). The raw RHPs were purified by drop precipitation into pentane and subsequent dialysis against water (MWCO 3000 Da). The raw products after polymerization, precipitation supernatants, dialysate solutions, and purified RHPs were collected and analyzed using NMR and GPC (Figures 3b,c, S8, S15, S22, and S26–S29). Minimal batch-to-batch variations were observed among triplicate batches.

### Trajectory of RHP Compositions during the Entire Preparation Process

**Figure 4:**
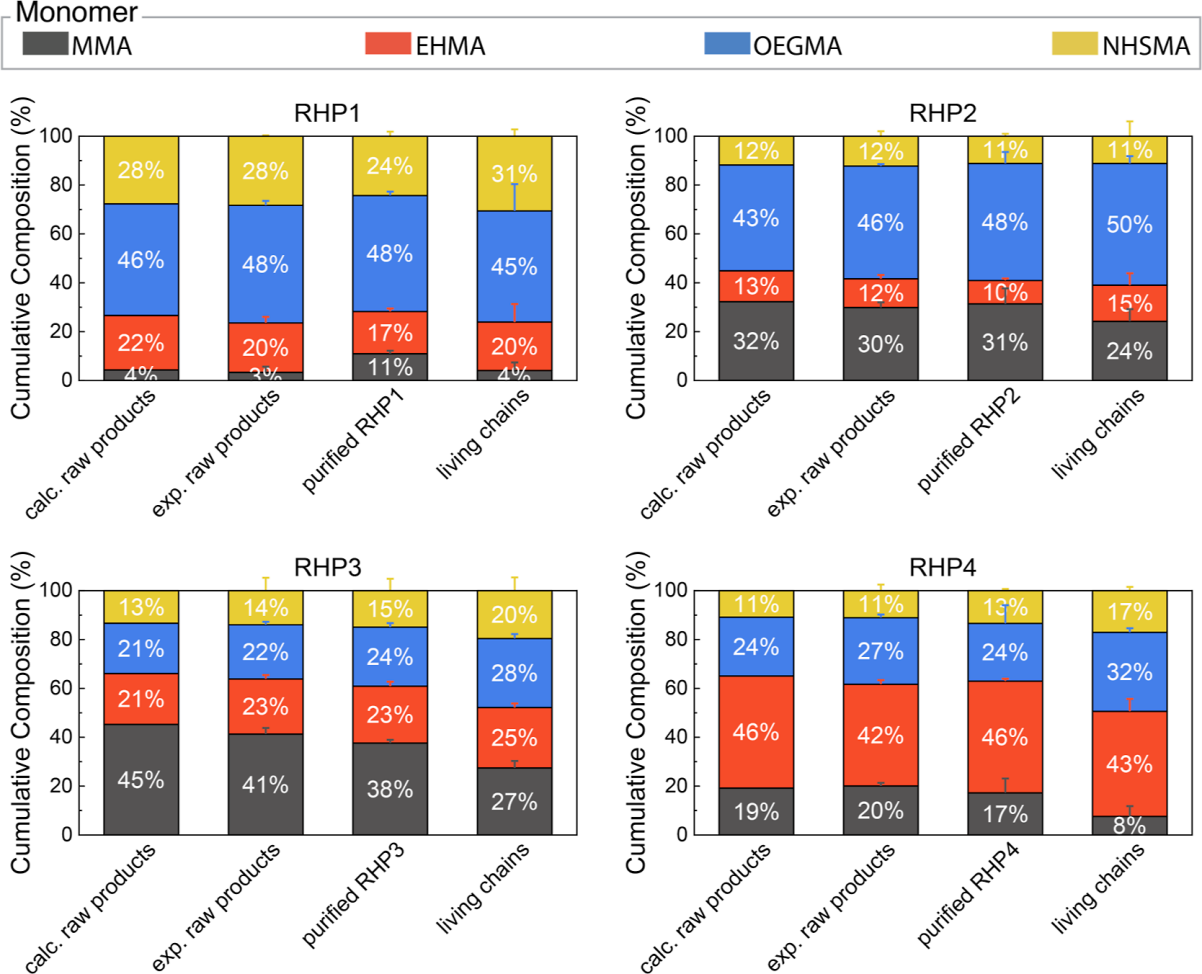
RHP composition during RAFT polymerization and purifications. The percentage represents molar percent composition, and the error bars represent deviation from three independent RHP synthesis batches. The simulation results represent averaged composition of 100,000 simulated individual chains. The compositions of living chains were calculated from at least nine depolymerization experiments.

We first investigated the composition drift during the RHP RAFT polymerization. The compositions of raw RHP products after RAFT polymerization were determined by quantifying the conversion of each monomer using ^1^H NMR from the initial feedstock and post-polymerization aliquots, assuming that all consumed monomers participated in the polymerization (Section S1 and Table S7).^36,60^ Upon analysis, we noticed a decrease in the total peak integral when we compared the ^1^H NMR spectra of the initial feedstocks with the post-polymerization aliquots. This decrease indicated a loss of monomers during the freeze–pump–thaw degassing process, which we attributed to the evaporation of volatile MMA under vacuum. A loss of approximately 20% in MMA was identified when contrasting the NMR spectra of the initial feedstock and the post-polymerization aliquots. To accurately estimate the composition of the raw products, this loss of MMA was factored into our calculations in determining the raw RHP compositions. With this correction, the experimental findings closely match the simulated results using experimental inputs, with a deviation of less than 4% (Figure 4 and Table S8). We conjecture that these deviations are likely due to the formation of dead chains during RAFT polymerization, which is currently not considered in the sequence simulation used here.^48^ Despite this, these results reinforce the validity of in silico RHP design by using reactivity ratios determined from quaternary copolymerization.

We observed distinct impacts of pentane precipitation and dialysis on the **RHP1–4** ensembles. GPC analysis shows that pentane precipitation eliminated chains from **RHP1**, **3**, and **4** (Figures 3c and S28 and S29). Conversely, dialysis against water removed chains from more hydrophilic **RHP1–2**, with minimal observable effects on less hydrophilic **RHP3–4** (Figure S26 and S27). These observations support our hypothesis that the post-polymerization purification processes can eliminate RHP chains, contingent on their apparent hydrophobicity.

**Figure 5:**
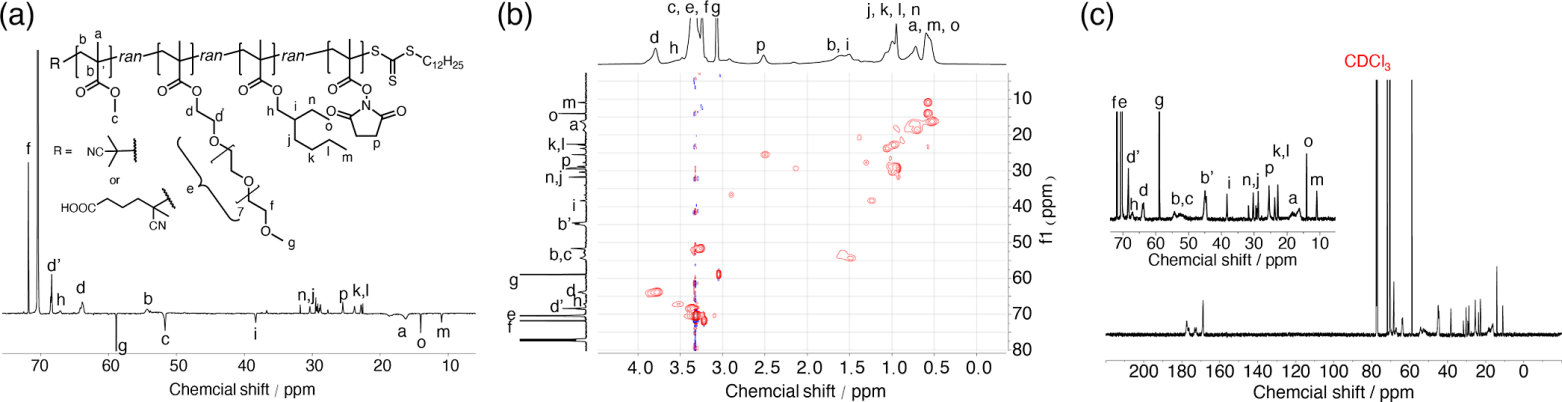
DEPT-135 ^13^C (a), ^1^H–^13^C HSQC (b), and quantitative ^13^C NMR (c) of purified **RHP1** (CDCl_3_).

Quantifying the compositions of eliminated RHP chains in precipitation supernatants and dialysate solutions was challenging because their signals were generally low in the NMR spectra and obscured by the residual monomers (Figures 3b, S8, S15, and S22). To better quantify the impacts of purification process, we compared the compositions of raw and purified RHPs. Considering the peak broadening of polymers in ^1^H NMR, we used DEPT-135 ^13^C and ^1^H–^13^C HSQC NMR experiments to identify the characteristic ^13^C signals of each monomer in purified RHPs, followed by studies using quantitative ^13^C NMR (Figures 5, S1–S24, and Table S9). Comparing raw RHP products with the purified RHPs, there is a minimal shift in composition (<4%), with the MMA fraction of **RHP1** exhibiting the largest shift (7%) (Figure 4). These observations are consistent with GPC analysis, which shows that only a minor fraction of RHP chains were removed during purification. Within the systematic errors in quantitative NMR analysis,^61^ these findings imply that despite the removal of partial RHP chains during the purification process, the overall composition and sequence heterogeneity remain largely unaffected.

**Figure 6:**
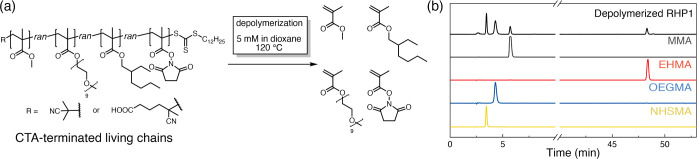
Reproposing chain-end depolymerization for quantifying RHP living chain composition (a) RHP chain-end unzipping depolymerization. (b) HPLC traces of depolymerized **RHP1** and corresponding monomers. Residual solvent peak (dioxane) is shown at 2.6 min.

To examine the statistical control obtained in the four-monomer RHP RAFT copolymerization, we further assessed the composition of RHP living chains. We applied a chain-end unzipping approach using a recently reported catalyst-free polymethylacrylate depolymerization method (Figure 6a).^62,63^ The terminal C–S bonds of living chains undergo thermal homolysis to trigger chain-end depolymerization, whereas the dead chain remains intact due to the lack of CTA end-groups. After depolymerization, the regenerated monomers from living chains were directly quantified by HPLC to determine the composition of RHP living chains (Figures 6b and S31, additional discussions in Section S4).

Analysis of the compositional differences denotes that the molar percent of MMA in RHP living chains is 7–9% lower compared to the corresponding purified RHPs (Figure 4 and Table S10). These results indicate that the dead chains within **RHP1–4** are more MMA-rich compared with the living chains. In a recent study, Zhang revealed that a larger fraction of dead chains are generated at the early lower conversion stages of RAFT polymerization due to the higher abundance of primary free radicals at the initial stages of the reaction.^64,65^ Likewise, as monomer conversion and chain length increase, a significant reduction in the termination rate coefficient, exceeding 3 orders of magnitude, was observed in the radical polymerization of MMA.^65,66^ Furthermore, prior work has reported that the termination rate coefficient of MMA in radical polymerization is 1 order of magnitude higher than that of methacrylates with longer side chains such as butyl and dodecyl methacrylate.^67,68^ In light of these results, the higher fraction of MMA in RHP dead chains can be ascribed to (1) the majority of RHP dead chains being formed during the early stages of polymerization, (2) during which RHP chains with a higher fraction of MMA are generated as suggested by stochastic simulation (Figure S38), due to their high reactivity (*r*_non-MMA monomer/MMA_ < 1, Figure 1b), and (3) the susceptibility of MMA-terminated chains to termination. Collectively, these findings represent an initial proof that the living and dead chains in RHPs may exhibit different composition distributions. For future efforts in synthesizing block heteropolymers through chain-end extension, we advocate for a thorough composition analysis that considers the compositional differences between the living and dead chains when analyzing the final products.

### Probing Chain Conformation Distribution Using High-Throughput SCFT Calculation

The sequence heterogeneity in RHP ensembles demands new approaches for mapping sequence to behavior at the single chain level. While atomistic molecular dynamics (MD) simulations are powerful to study the behaviors of a single RHP chain, it remains a formidable task to simulate a large number of chains across a wide sequence range.^69^ This is primarily due to the significant computational costs involved in robust statistical analysis. To exhaustively explore the sequence space, 2D lattice model has been developed for protein-like heteropolymers.^70,71^ However, this simplification restricts the accuracy of modeling complex 3D chain conformations. Therefore, it is necessary to develop new, high-throughput approaches without compromising insights into how sequence heterogeneity dictates property dispersity. To this end, we used a polymer self-consistent field theory (SCFT) to compute the single-chain conformation of coarse-grained RHPs (modeling details in Section S1).^72−75^ This theoretical approach is widely applicable to various polymer structures beyond the RHP system described here.

**Figure 7:**
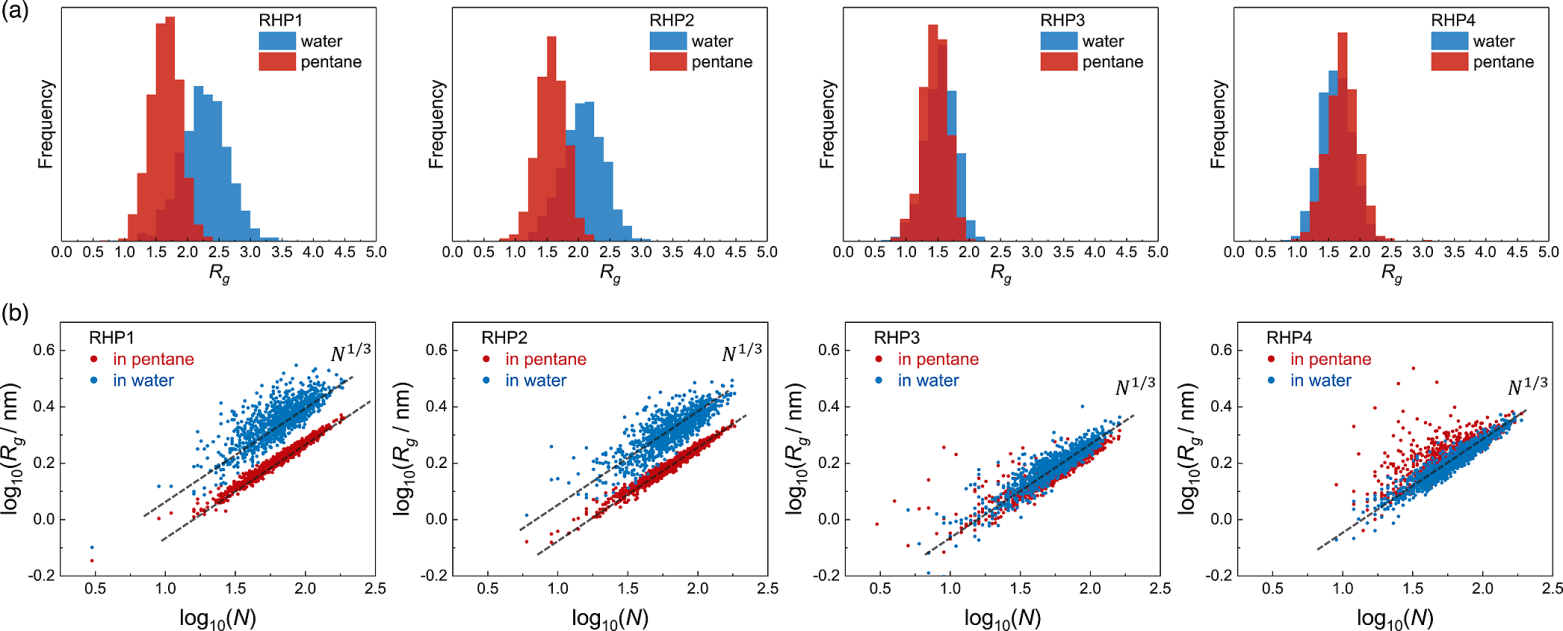
(a) Distribution of radius of gyration (*R*_g_/nm) for **RHP1–4** ensembles in pentane and water. (b) Scaling of *R*_g_ relative to chain length (*N*). Each dot represents an RHP chain with a unique sequence.

To facilitate statistical analysis, radius of gyration (*R*_g_) of a single chain was computed using 1000 sequences within each RHP ensemble. These sequences were randomly selected from the pool of 100,000 simulated chains for each model RHP. We studied the RHP chain conformation in two distinct solvent environments: water and pentane. Water is the prevalent solvent environment for applications involving protein-like RHPs, while pentane represents a nonpolar solvent environment.^19^ Both are also used in the RHP purification process. The computation results indicate that the scaling of radius of gyration (*R*_g_) relative to chain length (*N*) generally follows a *R*_g_ ∼ *N*^1/3^ correlation (Figure 7b), suggesting that RHP chains adopt an approximately globular conformation via chain-collapsing when exposed to water or pentane.^76^ This is in agreement with the atomistic MD simulation of RHPs.^77^ Yet, the studies provide insights into chain conformation distributions across the sequence space of RHPs as a function of monomer composition and segmental characteristics. **RHP1–4** exhibit similar *R*_g_ distribution in pentane (∼1–2.2 nm) (Figure 7a). However, in water, **RHP1–2** display a larger average *R*_g_ and wider distribution (∼1–3.2 nm) than **RHP3–4** (∼1–2.2 nm). In comparison to **RHP3–4**, **RHP1–2** chains adopt extended conformations with a higher degree of conformational heterogeneity in an aqueous environment. These findings are consistent with our design, wherein the average RHP chain hydrophobicity progressively increases from **RHP1** to **RHP4** (Figure 2c).

**Figure 8:**
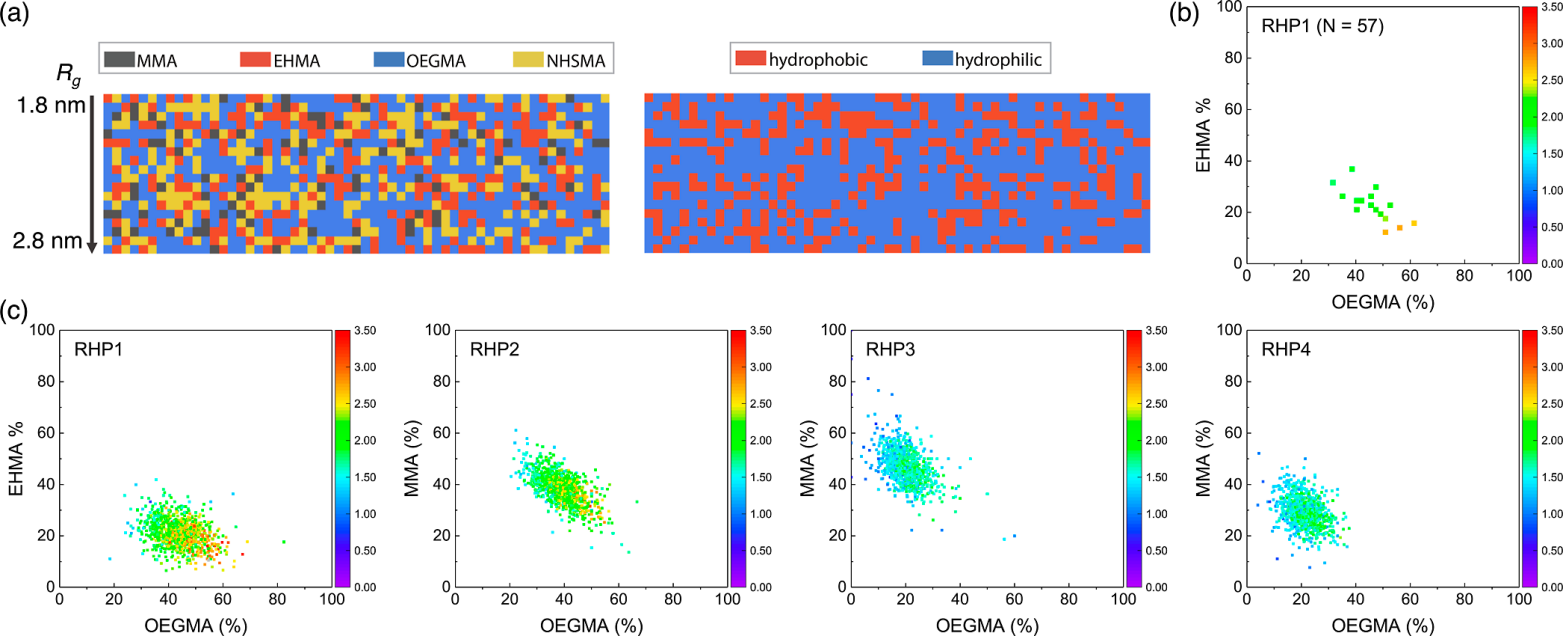
(a) Sequences with identical chain length (*N* = 57) sampled from the simulated **RHP1** ensemble. The sequences presented are organized in the ascending order of *R*_g_ (water). (b) Correlation between monomer composition and *R*_g_ (water) in sampled **RHP1** sequences. Scale bar represents the corresponding radius of gyration *R*_g_ (nm) in water. (c) Correlation between the monomer composition and *R*_g_ (water) in **RHP1–4** ensembles.

The high-throughput computational approach also allows us to backtrack sequences based on *R*_g_ and discern the major factors influencing *R*_g_. Taking the average chain length (*N* = 57) as a case study, we identified the RHP chains with identical chain length (*N* = 57) and sorted these sequences based on their *R*_g_ (Figures 8a and S37). This facilitates the sequence analysis by eliminating chain length dispersity and lowering the overall heterogeneity. In general, each of the four monomers is spread throughout the entire chain without long consecutive monomer blocks (>5). Notably, a strong connection is observed between the RHP chain conformation and composition (Figure 8b). In an aqueous environment, there is a notable rise in *R*_g_ (∼1 nm) as the fraction of OEGMA increases. This can be attributed to the hydrophilic nature of the OEGMA and its bulky side groups.

To further examine the potential of tailoring monomer composition for fine-tuning the resulting conformational distributions, we projected the whole *R*_g_ distributions of **RHP1–4** (in water) onto two representative monomers (MMA and OEGMA) (Figure 8c). **RHP1** differs from **RHP2–4** by its distinctive feature of having a minimal MMA ratio of only 10%. We therefore chose to project the *R*_g_ distribution of **RHP1** onto EHMA (composition ratio: 20%) and OEGMA (composition ratio: 45%) to better elucidate the composition-*R*_g_ correlation. This analysis enables us to identify the distinctions in the morphology characteristics across a broader composition space. The results reveal that in an aqueous environment, the RHP chain exhibits a higher *R*_g_ and more extended conformation when the OEGMA fraction is increased and the EHMA or MMA fraction is reduced. Given the widespread use of statistical heteropolymer ensembles in functional materials, the key design challenge has been how to tailor chain distributions for specific properties.^18,44,45^ Currently, the precise control of sequence in multimonomer copolymerization and the sequencing of synthetic polymers pose ongoing challenges.^78−80^ The findings presented here reveal that, despite the sequence heterogeneity, tuning the overall monomer composition can effectively shift the chain conformation distributions of statistical RHP ensembles. With careful monitoring of the livingness in multimonomer copolymerization and rigorous characterization of heteropolymer composition, this study showcases that in silico design based on ensemble-averaged composition and experimentally determined reactivity ratios can effectively guide the design of RHPs.

## Conclusions

In summary, we have presented a systematic study that serves as a basis for designing multicomponent RHPs. The approach combines experimental synthesis and characterization as well as coarse-grained computation. The methacrylate-based RHPs serve as a model system to demonstrate the process of determining the monomer reactivity ratios, quantifying polymer composition, selectively depolymerizing living chains for composition determination, simulating sequence, and understanding the composition–conformation correlations. These approaches collectively ensure that the multicomponent polymeric system can be constructed in a facile, systematic manner, expanding beyond the structures outlined here.

Moreover, understanding the compositional differences between the dead and living chains provides new insights into the details of the RAFT polymerization kinetics. As automated systems for polymer synthesis become increasingly accessible for RHP production, the necessity for strategically designing statistical RHP ensembles to achieve target properties is critical.^45,81^ There are potentially over 300 commodity methacrylate monomers that are available for synthesizing RHP materials.^82^ The systematic methodology presented here will become invaluable for designing fit-for-purpose RHPs. Furthermore, the results presented highlight the importance of composition in predicting chain conformation. When well-controlled polymerization is achieved to ensure statistical randomness in sequence control and minimal changes in overall composition, prioritizing composition emerges as a key design parameter for future analysis of RHP materials employing machine learning.

## Supporting Information Available

Materials and methods; sequence simulation procedure; modeling based on SCFT; RAFT polymerization and thermal-triggered depolymerization procedure; NMR spectra; GPC and HPLC results; determination of monomer reactivity ratios; summary of composition ratios; and additional sequence analysis (PDF)
