# Supplementary material for: Mapping Composition Evolution through Synthesis, Purification, and Depolymerization of Random Heteropolymers
Source: J Am Chem Soc. 2024 Feb 22;146(9):6178–88. doi: 10.1021/jacs.3c13909 (PMC10921401; doi:10.1021/jacs.3c13909)
Supplement: Supplementary file 1 — ja3c13909_si_001.pdf [file ja3c13909_si_001.pdf]

Supplementary Information for

Mapping Composition Evolution through Synthesis, Purification, and Depolymerization of Random Heteropolymers

Hao Yu[1], Luofu Liu[2], Ruilin Yin[3], Ivan Jayapurna[4], Rui Wang[2,5], and Ting Xu[1,3,4,5]*

[1]California Institute for Quantitative Biosciences, University of California, Berkeley, Berkeley, California 94720, United States

[2]Department of Chemical and Biomolecular Engineering, University of California, Berkeley, Berkeley, California 94720, United States

[3]Department of Chemistry, University of California, Berkeley, Berkeley, California 94720, United States

[4]Departent of Materials Science and Engineering, University of California, Berkeley, Berkeley, California 94720, United States

[5]Materials Science Division, Lawrence Berkeley National Laboratory, Berkeley, California 94720, United States

[*]To whom correspondence should be addressed.
Email: tingxu@berkeley.edu

Contents:

1. General Methods S2
2. RAFT Polymerization and Thermal-triggered Depolymerization S8
3. NMR Spectra S9
4. GPC and HPLC Results S23
5. Determining Monomer Reactivity Ratios S29
6. Summary of Composition Ratios S31
7. Additional Analysis S32
8. References S36

S.1 General Methods

Materials

All reagents were purchased from commercial suppliers and used as received unless otherwise stated. Azobisisobutyronitrile (AIBN) was recrystallized in methanol prior to use. Prior to polymerization, methyl methacrylate (MMA), 2-ethylhexyl methacrylate (EHMA), and ethylene glycol methyl ether methacrylate (OEGMA) were passed over a column of neutral alumina to remove inhibitors.

Nuclear Magnetic Resonance (NMR) Spectroscopy

^{1}H and ^{13}C NMR spectra (500 MHz and 150 MHz) were recorded at room temperature (298 K). Chemical shifts are reported in δ (ppm) referenced on residual solvent peaks. Coupling constants (J) are expressed in Hertz (Hz). Splitting patterns are designated as: s (singlet), d (doublet), t (triplet), and m (multiplet).

Determining Composition of Raw RHP Products

The consumption of each monomer during RAFT copolymerization was estimated by comparing the NMR spectra of feedstock and post-polymerization aliquots (using DMF peak as internal standard). The initial feedstock NMR aliquot was taken before the degassing process. The analysis revealed that a decrease in the total peak integral between the feedstock and post-polymerization NMR spectra, indicating the loss of monomers during the freeze-pump-thaw degassing procedure. This can be attributed to the loss of volatile MMA during the degassing process. Therefore, we estimated the loss of MMA by comparing the NMR spectra of initial feedstock and post-polymerization aliquots. We factored this loss into our calculations when determining the composition of the raw RHP. This calibration allows us to obtain an accurate estimation of the raw product composition.

Determining Composition of Purified RHP Products

Quantitative ^{13}C NMR was performed using an inverse-gated decoupling sequence ("zgig" on a Bruker Avance III instrument equipped with a 5 mm 1H/BB Prodigy cryo-probe) with a 60s relaxation delay (150 MHz, $CDCl_3$). RHPs monomer composition ratios were determined by integrating the peaks labeled with c (MMA), d (OEGMA), h (EHMA), p (NHSMA) and calculating the fraction for each monomer.

Gel Permeation Chromatography (GPC)

THF GPC data were collected via an instrument equipped with an isocratic pump (1260 Infinity II, Agilent) and a differential refractometer (dRI) detector. Size exclusion columns were maintained at a temperature of 40 ºC using THF as the mobile phase. All sample solutions were filtered through a 0.45 μm PTFE filter before injection. The molecular weight of each polymer was determined based on a PMMA calibration curve.

DMF GPC data were collected using a Malvern OmniSEC equipped with refractive index, light scattering, and intrinsic viscosity detectors calibrated with a single PMMA standard. Analysis was performed in dimethylformamide containing 0.2% lithium bromide running at 0.7 mL/min and 55 ºC. All sample solutions were prepared at a concentration of 2 mg/ml and filtered through a 0.45 μm PTFE filter before injection.

Spin Dialysis and Lyophilizing

Spin dialysis was performed using MilliporeSigma Amicon Ultra-15 Centrifugal Filter Units (Part number: UFC900324), diameter: 29.7 mm, MWCO: 3,000 d, volume: 15 mL. After

pentane precipitation, RHPs were dissolved in 10 mL DI water and transferred to the filter unit. The mixture was centrifuged at 6000 rpm for 30 min until the remaining solution was around 2 mL. 10 mL DI water was then added to the remaining mixture and the centrifuge separation was repeated. A total of 30 mL dialysate was collected for each RHP, and no residual monomers were observed in the NMR analysis of the final products. After dialysis, the purified products were dissolved in water to make a concentrated solution and transferred into vial for lyophilizing (using a 8L LABCONCO FreeZone Freeze Dryers). The purified RHPs were then characterized by NMR and GPC and used in depolymerization experiments.

High Performance Liquid Chromatography (HPLC)

The monomer composition ratio after depolymerization were determined using a RP-HPLC (Agilent) on a C4 column (Viva 5 µm, 200 mm x 4.6 mm). The flow rate was 1 ml/min. Elution was monitored with a diode array detector at wavelengths of 210 nm. When measuring the UV-vis spectra of the monomers (MMA, EHMA, OEGMA, and NHSMA), no significant absorbance was observed above 220 nm. The selection of a 210 nm detection wavelength was based on its high sensitivity to detect ester compounds.[1] Elution gradient: 20 min 20%IPA/80%water plus 0.1% (v/v) TFA then a 30 min linear AB gradient from 0% B to 50% B, where solvent A consisted of 20%IPA/80%water plus 0.1% (v/v) TFA and solvent B consisted of acetonitrile plus 0.1% (v/v) TFA.

Composition and Sequence Simulation

Composition and sequence simulations of the synthesized RHPs were performed using a recently developed open-source software *RHPapp* (https://www.ocf.berkeley.edu/~xugroup/rhpapp/).[2] The simulation inputs (including the monomer composition, RAFT to initiator ratio, conversion fraction, and average degree of polymerization) were based on experimental design. A total of 100,000 RHP sequences were generated for each model RHP batch to ensure that there are sufficient chains to yield composition distributions for statistical analysis.

Hydrophobicity Calculation and Statistical Analysis

The RHP chain hydrophobicity was calculated based on previously reported hydrophilic-lipophile balance (HLB) scale estimated by considering the functional groups on the monomer side chains.[3] The four monomers used in this experiment and simulation were each assigned a HLB index, where MMA was assigned a value of 8.45, EHMA a value of 5.125, OEGMA a value of 11.42, and NHSMA a value of 12.775.[2,4,5] The hydrophobicity of each chain of the RHP and the distribution of that HLB value were calculated based on value assignment above.

Self-consistent Field Theory

1. **Model and Theory**

We develop a theoretical model for RHPs to calculate their single-chain conformations in solvents. The system we consider is a subvolume V consisting of one RHP chain and the surrounding n_s solvent molecules. The system is treated as a semicanonical ensemble: the number of RHP chain is fixed to be 1 whereas the number of solvent molecules is controlled by a reservoir with fixed solvent chemical potential μ_s. As shown in the figure below, the RHP is modeled by a discrete Gaussian chain with $(N+1)$ beads sequentially connected by N gaussian springs with a Kuhn length b. Each bead stands for a monomer in one of the four types (MMA, OEGMA, EHMA and NHSMA) in the RHP sequence. The RHP sequences are obtained following the procedures listed under the section of "Composition and Sequence Simulation". The volume of the i-th monomer on the RHP chain is denoted by v_i and the solvent volume is denoted by v_s. The non-bonded interactions between different monomers as

well as between monomers and solvents are described by Flory-Huggins (F-H) parameters. Specifically, χ_{ij} is the F-H parameter between the i-th and j-th monomer and χ_{is} is that between the i-th monomer and the solvent.

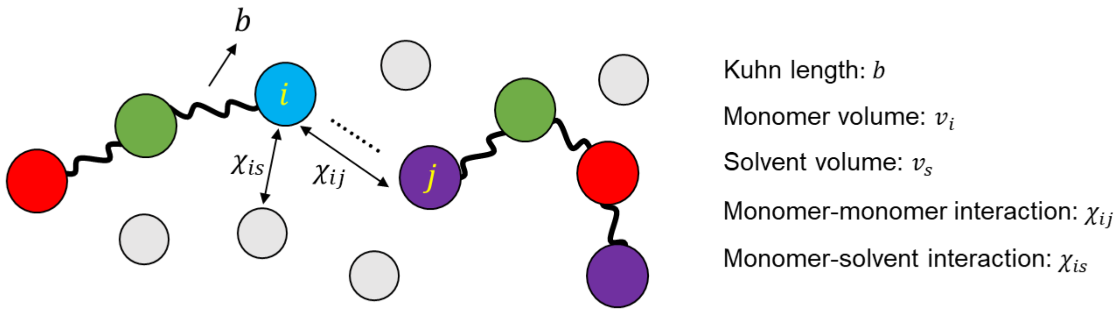

Scheme S1. Schematic of the coarse-grained model for RHPs. The RHP chain is modeled by a discrete Gaussian chain with beads sequentially connected by springs with Kuhn length b. Each colored bead stands for a monomer in one of the four types (MMA, OEGMA, EHMA and NHSMA). The gray beads represent solvent molecules. The non-bonded interactions between different types of monomers as well as between monomers and solvent molecules are described by Flory-Huggins parameters χ_{ij} and χ_{is}.

The Hamiltonian of the system includes both the bonded and non-bonded interactions, given by

$$\beta H = \sum_{i=0}^{N-1} \frac{3}{2b^2} (\boldsymbol{R}_{i+1} - \boldsymbol{R}_i)^2 + \frac{1}{v_0} \int \mathrm{d}\boldsymbol{r} \left[\sum_{i=0}^{N} \chi_{is} \hat{\phi}_i(\boldsymbol{r}) \hat{\phi}_s(\boldsymbol{r}) + \frac{1}{2} \sum_{i=0}^{N} \sum_{j=0}^{N} \chi_{ij} \hat{\phi}_i(\boldsymbol{r}) \hat{\phi}_j(\boldsymbol{r}) \right] \quad (1)$$

where $\boldsymbol{R}_i$ is the position vector of the i-th monomer on the RHP chain. v_0 is a reference volume for F-H interaction parameters. $\hat{\phi}_i(\boldsymbol{r}) = v_i \delta(\boldsymbol{r} - \boldsymbol{R}_i)$ and $\hat{\phi}_s(\boldsymbol{r}) = v_s \sum_{\kappa=1}^{n_s} \delta(\boldsymbol{r} - \boldsymbol{r}_\kappa)$ are the instantaneous volume fractions of the i-th monomer and the solvent, respectively. The partition function of the semicanonical ensemble is then given by

$$Z = \sum_{n_s=0}^{\infty} \frac{e^{\beta \mu_s n_s}}{n_s! \, v_0^{n_s+N+1}} \int \mathrm{D}\boldsymbol{R} \prod_{\kappa=1}^{n_s} \int \mathrm{d}\boldsymbol{r}_\kappa \prod_{\boldsymbol{r}} \delta \left[1 - \hat{\phi}_s(\boldsymbol{r}) - \sum_{i=0}^{N} \hat{\phi}_i(\boldsymbol{r}) \right] \exp(-\beta H) \quad (2)$$

where $\int \mathrm{D}\boldsymbol{R}$ denotes a functional integration over all possible chain conformations of the RHP, $\int \mathrm{d}\boldsymbol{r}_\kappa$ stands for the integration over the solvent degrees of freedom. The δ-functional accounts for the incompressibility. We perform the standard self-consistent field approach[6–8] which involves (1) decoupling this many-body problem into a single-chain interacting with fluctuating fields using identity transformations, and (2) replacing the functional integration over the fluctuating fields by the saddle point approximation. This procedure ultimately leads to the self-consistent equations to solve for the RHP conformation:

$$w_i(\boldsymbol{r}) = \chi_{is} \phi_s(\boldsymbol{r}) + \sum_{j=0}^{N} \chi_{ij} \phi_j(\boldsymbol{r}) + \xi(\boldsymbol{r}) \quad (3a)$$

$$w_s(\boldsymbol{r}) = \sum_{i=0}^{N} \chi_{is}\phi_i(\boldsymbol{r}) + \xi(\boldsymbol{r}) \tag{3b}$$

$$\phi_i(\boldsymbol{r}) = \frac{\tilde{v}_i}{Q_p} q(\boldsymbol{r}, i) \exp\big(\tilde{v}_i w_i(\boldsymbol{r})\big) q^*(\boldsymbol{r}, i) \tag{3c}$$

$$\phi_s(\boldsymbol{r}) = \tilde{v}_s \exp\big(\beta\mu_s - \tilde{v}_s w_s(\boldsymbol{r})\big) \tag{3d}$$

$$\sum_{i=0}^{N} \phi_i(\boldsymbol{r}) + \phi_s(\boldsymbol{r}) = 1 \tag{3e}$$

where w_i and w_s are the fields conjugate to the density of the i-th monomer and the solvent, respectively. ξ is the field corresponding to the incompressibility condition. $\tilde{v}_i = v_i/v_0$ and $\tilde{v}_s = v_s/v_0$ are the rescaled volumes of monomer and solvent. Q_p is the partition function of the RHP chain, given by

$$Q_p = \frac{1}{v_0}\int d\boldsymbol{r}\, q(\boldsymbol{r}, i) \exp\big(\tilde{v}_i w_i(\boldsymbol{r})\big) q^*(\boldsymbol{r}, i) \tag{4}$$

where the forward chain propagator q and the backward chain propagator q^* are determined by the following integral equations

$$q(\boldsymbol{r}, i+1) = \exp\big(-\tilde{v}_{i+1} w_{i+1}(\boldsymbol{r})\big) \int d\boldsymbol{r}'\, \Phi(\boldsymbol{r} - \boldsymbol{r}') q(\boldsymbol{r}, i) \tag{5a}$$

$$q^*(\boldsymbol{r}, i-1) = \exp\big(-\tilde{v}_{i-1} w_{i-1}(\boldsymbol{r})\big) \int d\boldsymbol{r}'\, \Phi(\boldsymbol{r} - \boldsymbol{r}')\, q^*(\boldsymbol{r}, i) \tag{5b}$$

with the initial condition $q(\boldsymbol{r}, 0) = \exp(-\tilde{v}_0 w_0(\boldsymbol{r}))$ and $q^*(\boldsymbol{r}, N) = \exp(-\tilde{v}_N w_N(\boldsymbol{r}))$. Φ is the Gaussian distribution function as $\Phi(\boldsymbol{r}) = \left(\frac{3}{2\pi b^2}\right)^{3/2} \exp(\frac{-3r^2}{2b^2})$. The free energy of the system is given by

$$\beta F = -\ln Q_p - e^{\beta\mu_s} Q_s + \frac{1}{v_0}\int \mathrm{d}\boldsymbol{r} \left[\sum_{i=0}^{N} \chi_{is}\phi_i\phi_s + \frac{1}{2}\sum_{i=0}^{N}\sum_{j=0}^{N} \chi_{ij}\phi_i\phi_j - \sum_{i=0}^{N} w_i\phi_i - w_s\phi_s\right] \tag{6}$$

where $Q_s = \frac{1}{v_0}\int d\boldsymbol{r}\, e^{-\tilde{v}_s w_s}$ is the solvent partition function.

Eqs. 3-5 are solved numerically to obtain the distribution of monomer and solvent volume fractions. The radius of gyration of the RHP is calculated by

$$R_g = \frac{\int \mathrm{d}\boldsymbol{r}\, \boldsymbol{r}^2 \phi(\boldsymbol{r})}{\int \mathrm{d}\boldsymbol{r}\, \phi(\boldsymbol{r})} \tag{7}$$

where $\phi = \sum_{i=0}^{N} \phi_i$ is the volume fraction of the whole RHP.

2. Parameterization

The volumes of monomer (v_i) and solvent (v_s) are calculated based on their molecular weights and densities, which are listed in **Table S1** below.

Table S1. Molecular weight, density, and volume of monomers and solvents.

	MMA	OEGMA	EHMA	NHSMA	Pentane	Water
Molecular weight (g/mol)	100.12	496.59	198.30	183.16	72.15	18.02
Density (g/cm^3)	0.944	1.070	0.880	1.290	0.626	1.000
Volume (nm^3)	0.177	0.770	0.374	0.236	0.193	0.030

The reference volume v_0 is taken to be the same value of the solvent volume. In addition, we use the Kuhn length $b = 0.5$ nm based on the statistical segment lengths of polymers formed by common alkene species.[9]

The F-H parameters between any two components i and j are estimated based on the Hansen solubility parameters with the following equation proposed by Lindvig *et al*[10]:

$$\chi_{ij} = 0.6\frac{v_0}{k_B T} * \left[\left(\delta_{D,i} - \delta_{D,j}\right)^2 + 0.25\left(\delta_{P,i} - \delta_{P,j}\right)^2 + 0.25\left(\delta_{H,i} - \delta_{H,j}\right)^2\right] \quad (9)$$

where δ_D, δ_P, and δ_H are the contributions from dispersion forces, polarization forces, and hydrogen-bonding effects. We list the solubility parameters for relevant species adopted from ref 9 in **Table S2.**[11]

Table S2. Hansen Solubility Parameters of monomers and solvents adopted from ref 9. "~" means the value is not directly accessible but estimated from that of similar structures.

	MMA	OEGMA	EHMA	NHSMA	Pentane	Water
δ_D (MPa$^{1/2}$)	18.6	17.0	~15.0	~17.0	14.5	15.5
δ_P (MPa$^{1/2}$)	10.5	10.7	~3.0	~15.0	0.0	16.0
δ_H (MPa$^{1/2}$)	7.5	8.9	~5.5	~15.0	0.0	42.3

The F-H parameters in both pentane and water calculated from Eq. 9 are listed in **Tables S3** and **S4**.

Table S3. Flory-Huggins interaction parameters in pentane.

	MMA	OEGMA	EHMA	NHSMA
MMA	0	0.086	0.789	0.611
OEGMA	0.086	0	0.611	0.392
EHMA	0.789	0.611	0	1.762
NHSMA	0.611	0.392	1.762	0
Pentane	1.645	1.540	0.283	3.344

Table S4. Flory-Huggins interaction parameters in water.

	MMA	OEGMA	EHMA	NHSMA
MMA	0	0.013	0.123	0.095
OEGMA	0.013	0	0.095	0.061
EHMA	0.123	0.095	0	0.274
NHSMA	0.095	0.061	0.274	0

Water	1.400	0.45	1.668	0.826

It should be noted that the F-H parameter between OEGMA and water reported in the previous experiment[12] ($\chi_{ij} \approx 0.45$) is much smaller than the value directly calculated from Eq. 9 ($\chi_{ij} = 1.261$). This might be caused by the well-ordered and tightly bounded water molecules in the side group of the PEG oligomer in OEGMA, which greatly enhances the hydrophilicity and lowers its F-H parameter with water.[13] Such effect cannot be fully captured by the solubility parameters. Therefore, in this work, we use $\chi_{ij} = 0.45$ for the OEGMA-water pair.

3. Numerical Details

Based on the symmetry of RHP chains in solvents, we use a spherical coordinate to perform numerical calculations. The box size L is taken to be 5 times the radius of gyration in the ideal state, i.e. $L = 5R_{g,id} = 5(Nb^2/6)^{1/2}$. The box is uniformly discretized into 200 grids such that the grid size is approximately $0.1b$, which guarantees sufficient spatial resolution of the RHP conformation. The equilibrium structure of the RHP can be obtained by solving Eqs. 3-5 iteratively until convergence. We start with an initial guess of volume fraction of the i-th monomer using a hyperbolic tangent function as $\phi_i(r) = (\tilde{v}_i/2)(1 - \tanh(\frac{r-R_0}{b}))$. The initial guess of the fields is then obtained by Eqs. 3a and 3b without the incompressible field ξ. We use the following strategy to update the fields. Fields conjugate to the volume fractions (w_i and w_s) are updated by a simple mixing rule, i.e., $w^{\text{new}} \leftarrow \alpha w^{\text{new}} + (1-\alpha) w^{\text{old}}$. The field conjugated to the incompressible condition is updated as $\xi^{new} \leftarrow \xi^{old} + \kappa * \max(|\sum \phi_i + \phi_s - 1|)$, where the second term on the r.h.s is used to enforce the incompressibility. In our calculation, we used the step sizes $\alpha = 0.02$ and $\kappa = 2$.

In spherical coordinates, Eq. 5 can be simplified as

$$q(r, i+1) = e^{-\tilde{v}_{i+1} w_{i+1}(r)} \int_0^\infty \mathrm{d}r' \frac{r'}{r} \left[e^{-\frac{3(r'-r)^2}{2b^2}} - e^{-\frac{3(r'+r)^2}{2b^2}} \right] q(r', i) \qquad (10a)$$

$$q^*(r, i-1) = e^{-\tilde{v}_{i-1} w_{i-1}(r)} \int_0^\infty \mathrm{d}r' \frac{r'}{r} \left[e^{-\frac{3(r'-r)^2}{2b^2}} - e^{-\frac{3(r'+r)^2}{2b^2}} \right] q^*(r', i) \qquad (10b)$$

The evaluation of forward and backward chain propagators is achieved by direct numerical integration with a cutoff value $2.5b$. This means, the integration is conducted only in the region $|r - r'| < 2.5b$. The contribution outside this range can be safely neglected due to the fast decay of the exponential function. This guarantees accuracy while reducing the computational burden.

The convergence criterion is that the change of free energy (relative to the obtained free energy from the previous iteration) is below 10^{-8}, and the error in the incompressibility condition $\max(|\sum \phi_i + \phi_s - 1|)$ is below 10^{-5}.

S.2 RAFT Polymerization and Thermal-triggered Depolymerization

General Procedures of RAFT Polymerization

A 20 mL Schlenk tube was charged with MMA, OEGMA, EHMA, NHSMA, and 2.5 mL DMF. 4-Cyano-4-[(dodecylsulfanylthiocarbonyl)sulfanyl]pentanoic acid, and AIBN were added, and the reaction mixture was degassed by three freeze-pump-thaw cycles. After stirring at 80 °C for a given time (**RHP1-2**: 2.5 hr; **RHP3-4**: 1.5 hr), the reaction mixture was cooled in liquid nitrogen and the polymer was precipitated by dropwise addition of the mixture into stirring pentane. The yellow precipitate was then dissolved in water and transferred to a 3000 MWCO dialysis bag. The polymer was dialyzed against water and lyophilized to yield the final product. The purified polymer was subsequently characterized using GPC, ^{1}H-NMR, and ^{13}C-NMR.

RHP-1: Yellow gel. Prepared using MMA (0.100 g, 1.0 mmol), OEGMA (2,236 g, 4.5 mmol), EHMA (0.396 g, 2.0 mmol), NHSMA (0.457 g, 2.5 mmol), 4-Cyano-4-[(dodecylsulfanylthiocarbonyl)sulfanyl]pentanoic acid (40 mg, 0.1 mmol), AIBN (3 mg, 0.02 mmol), and 2.5 mL DMF following the general RAFT polymerization procedures. Yield: 78 %.

RHP-2: Yellow gel. Prepared using MMA (0.402 g, 4.0 mmol), OEGMA (1.987 g, 4.0 mmol), EHMA (0.198 g, 1.0 mmol), NHSMA (0.183 g, 1.0 mmol), 4-Cyano-4-[(dodecylsulfanylthiocarbonyl)sulfanyl]pentanoic acid (40 mg, 0.1 mmol), AIBN (3 mg, 0.02 mmol), and 2.5 mL DMF following the general RAFT polymerization procedures. Yield: 62 %.

RHP-3: Yellow gel. Prepared using MMA (0.502 g, 5.0 mmol), OEGMA (0.994 g, 2.0 mmol), EHMA (0.396 g, 2.0 mmol), NHSMA (0.183 g, 1.0 mmol), 4-Cyano-4-[(dodecylsulfanylthiocarbonyl)sulfanyl]pentanoic acid (40 mg, 0.1 mmol), AIBN (3 mg, 0.02 mmol), and 2.0 mL DMF following the general RAFT polymerization procedures. Yield: 47 %.

RHP-4: Yellow gel. Prepared using MMA (0.251 g, 2.5 mmol), OEGMA (1.242 g, 2.5 mmol), EHMA (0.791 g, 4.0 mmol), NHSMA (0.183 g, 1.0 mmol), 4-Cyano-4-[(dodecylsulfanylthiocarbonyl)sulfanyl]pentanoic acid (40 mg, 0.1 mmol), AIBN (3 mg, 0.02 mmol), and 2.0 mL DMF following the general RAFT polymerization procedures. Yield: 45 %.

General Procedure of RHP Depolymerization

To a round-bottom flask with a stir bar was added RHPs and alumina-filtered dioxane (20 mg/20 mL). The reaction mixture was bubbled with N_2 gas for 30 min and stirred at 120 °C for a given time. After reaction, the mixture was cooled in liquid nitrogen and aliquot was took for characterizations.

S.3 NMR Spectra

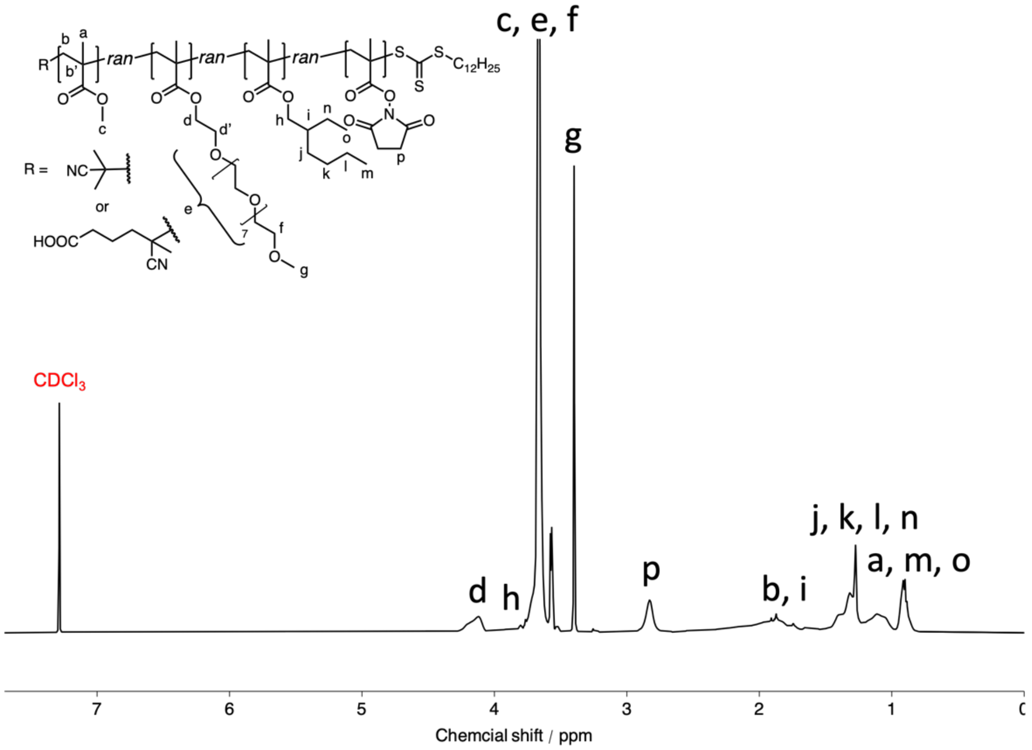

Figure S1. ^{1}H NMR spectrum of **RHP-1** (500 MHz, $CDCl_3$).

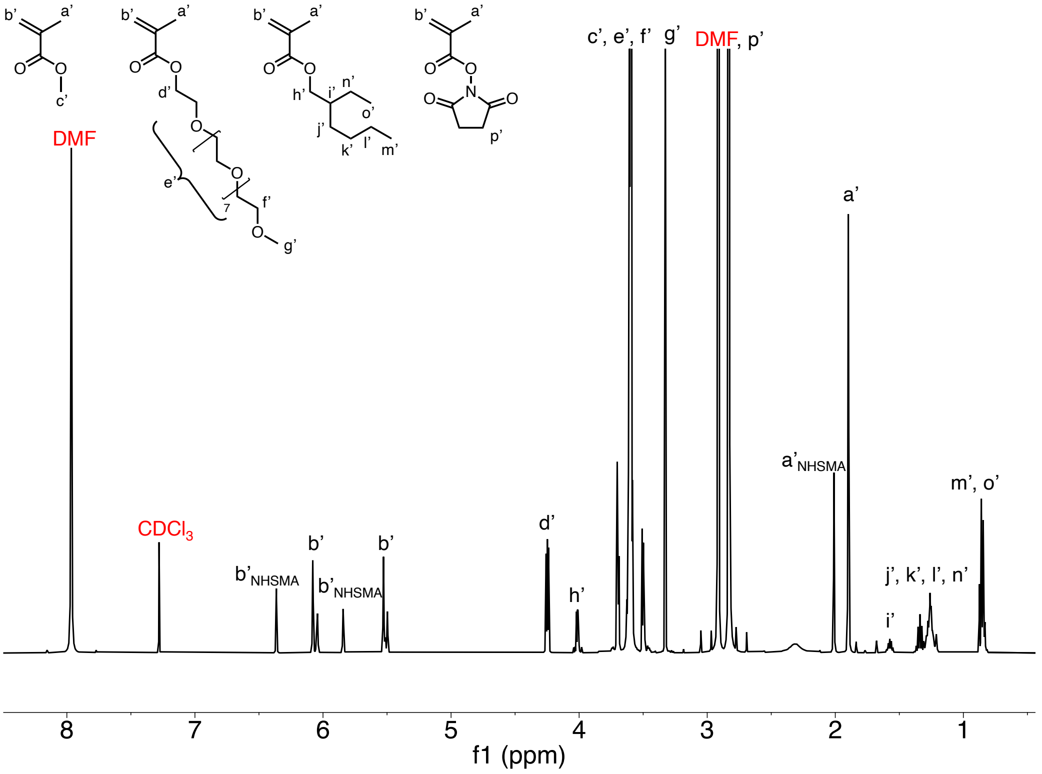

Figure S2. ^{1}H NMR spectrum of **RHP-1** pre-polymerization mixture (500 MHz, $CDCl_3$).

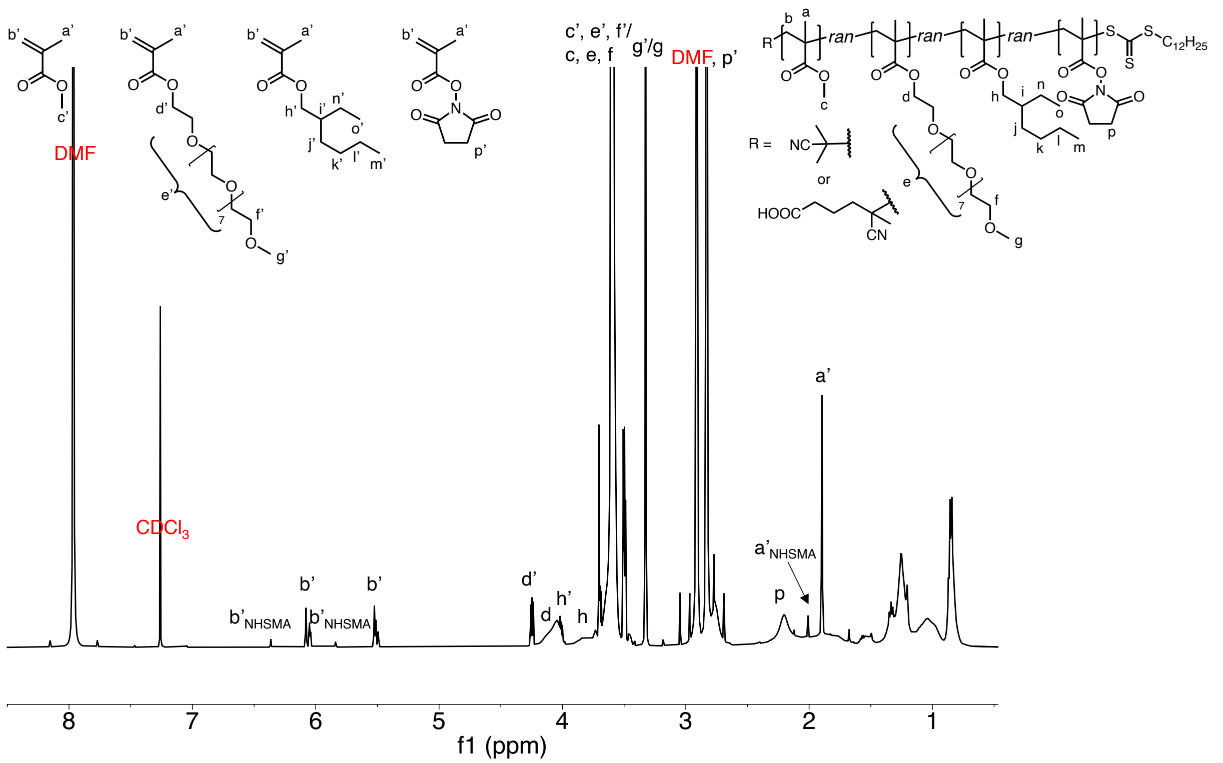

Figure S3. ^{1}H NMR spectrum of **RHP-1** post-polymerization mixture (500 MHz, $CDCl_3$).

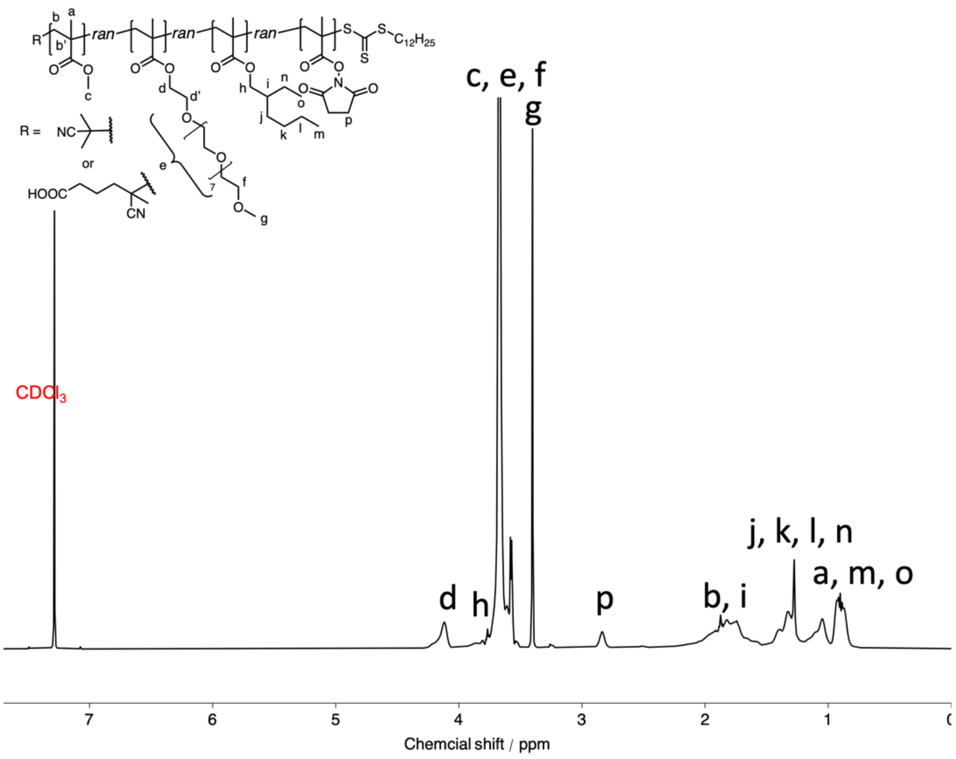

Figure S4. ^{1}H NMR spectrum of **RHP-2** (500 MHz, $CDCl_3$).

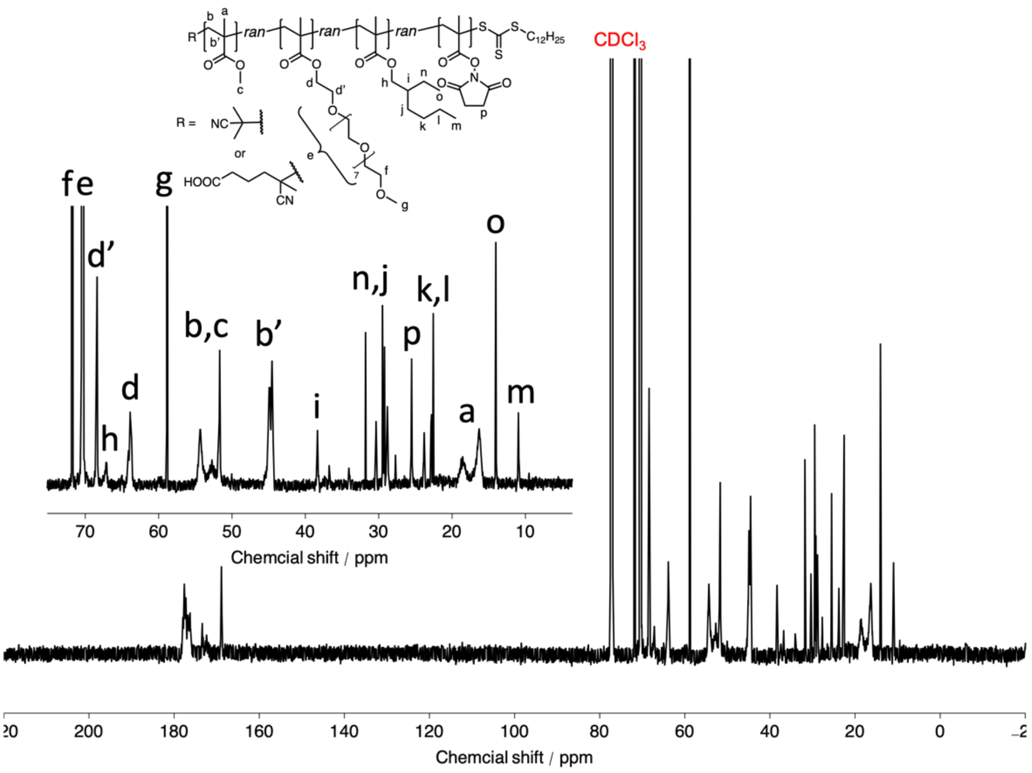

Figure S5. ^{13}C NMR spectrum of **RHP-2** (125 MHz, $CDCl_3$).

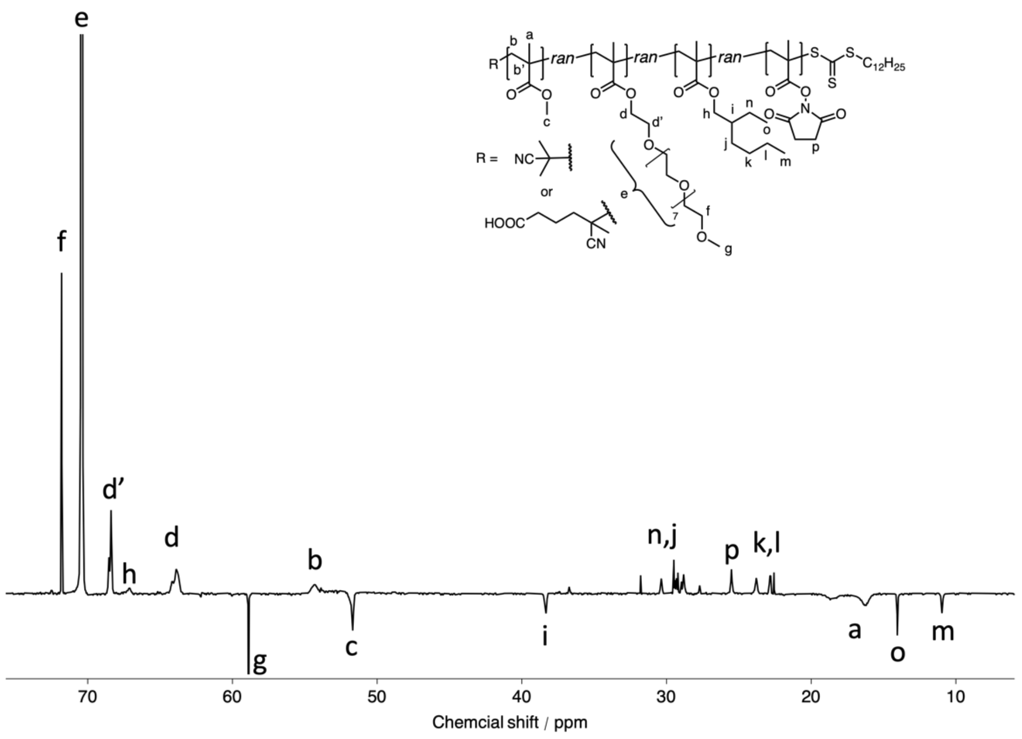

Figure S6. DEPT135 ^{13}C NMR spectrum of **RHP-2** (125 MHz, $CDCl_3$).

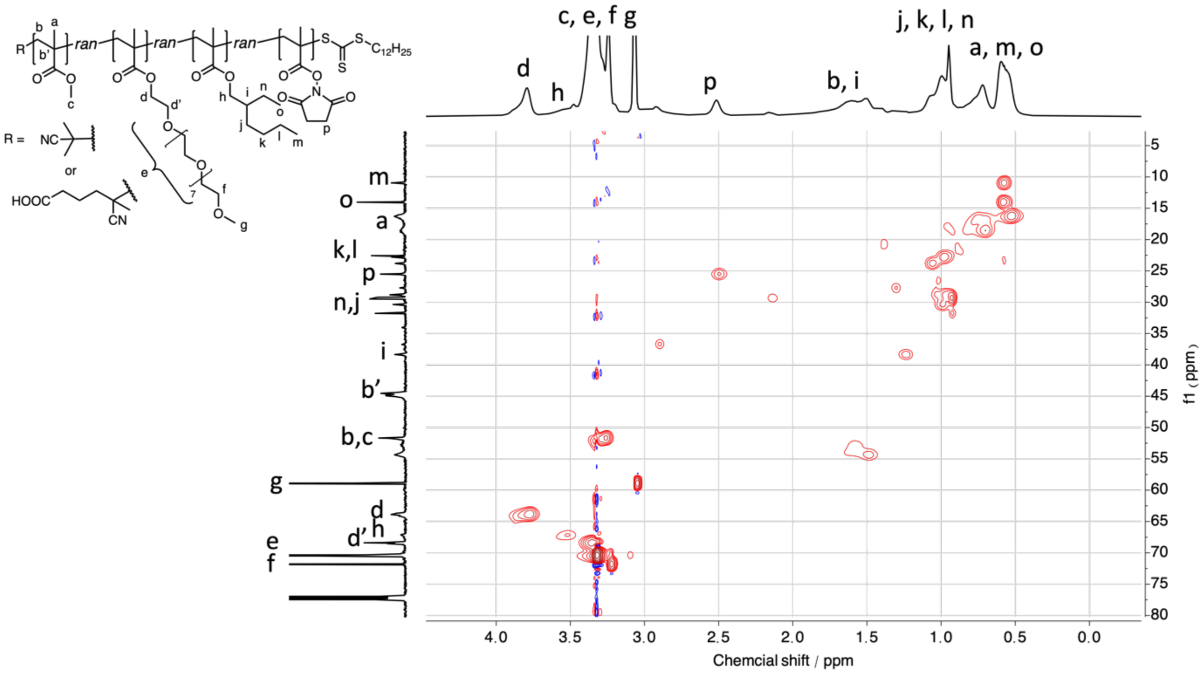

Figure S7. 2D ^{1}H-^{13}C HSQC NMR spectrum of **RHP-2** ($CDCl_3$).

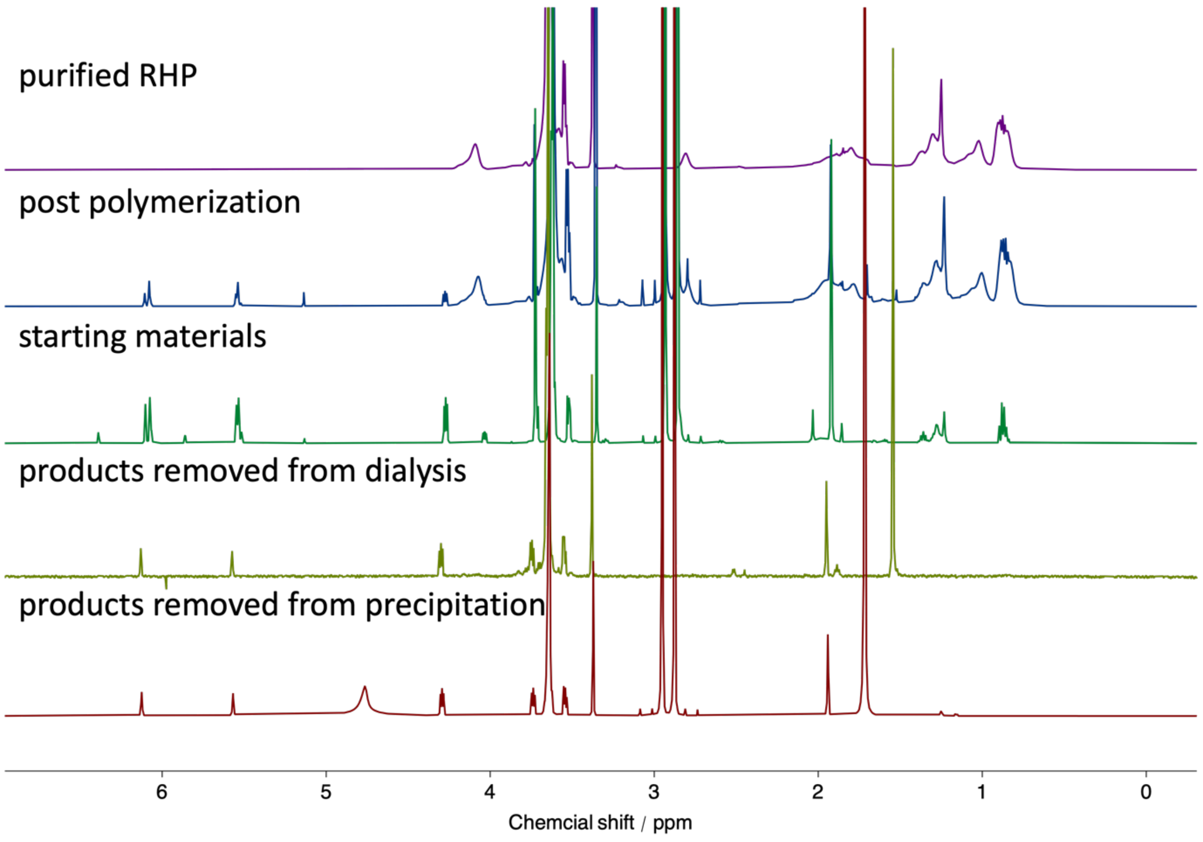

Figure S8. Comparison of ^{1}H NMR spectrum of **RHP-2**, crude products, starting materials, and products removed during purification (500 MHz, $CDCl_3$).

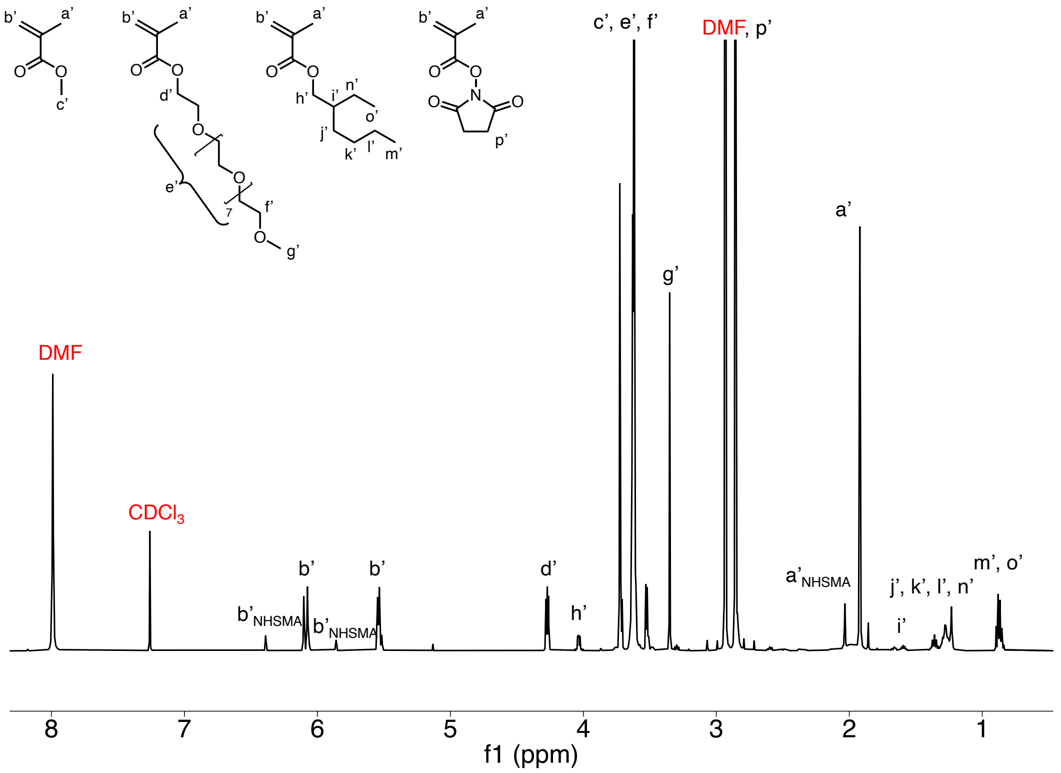

Figure S9. ^{1}H NMR spectrum of **RHP-2** pre-polymerization mixture (500 MHz, $CDCl_3$).

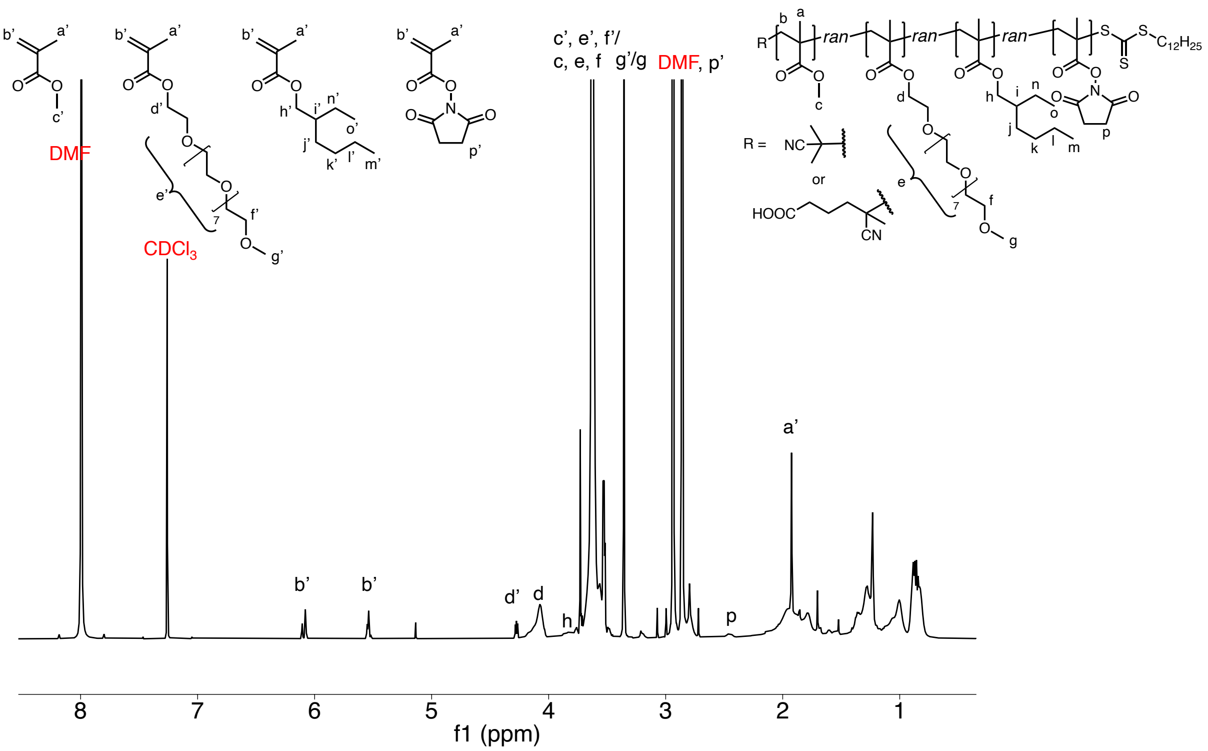

Figure S10. ¹H NMR spectrum of **RHP-2** post-polymerization mixture (500 MHz, $CDCl_3$).

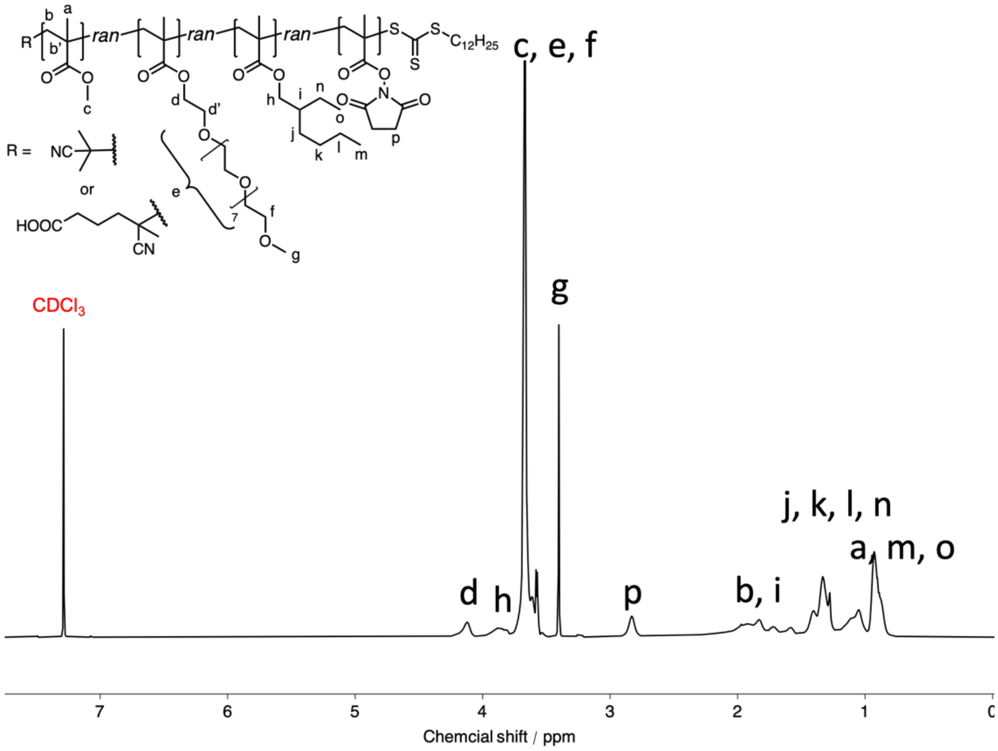

Figure S11. ^{1}H NMR spectrum of **RHP-3** (500 MHz, $CDCl_3$).

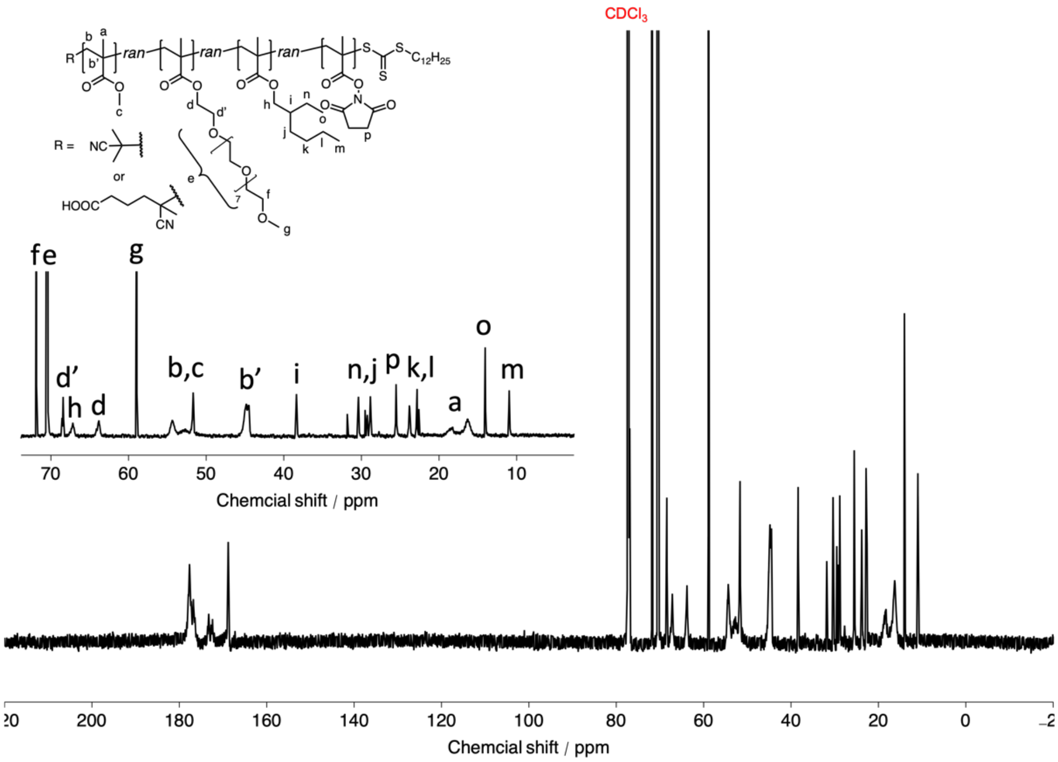

Figure S12. ^{13}C NMR spectrum of **RHP-3** (125 MHz, $CDCl_3$).

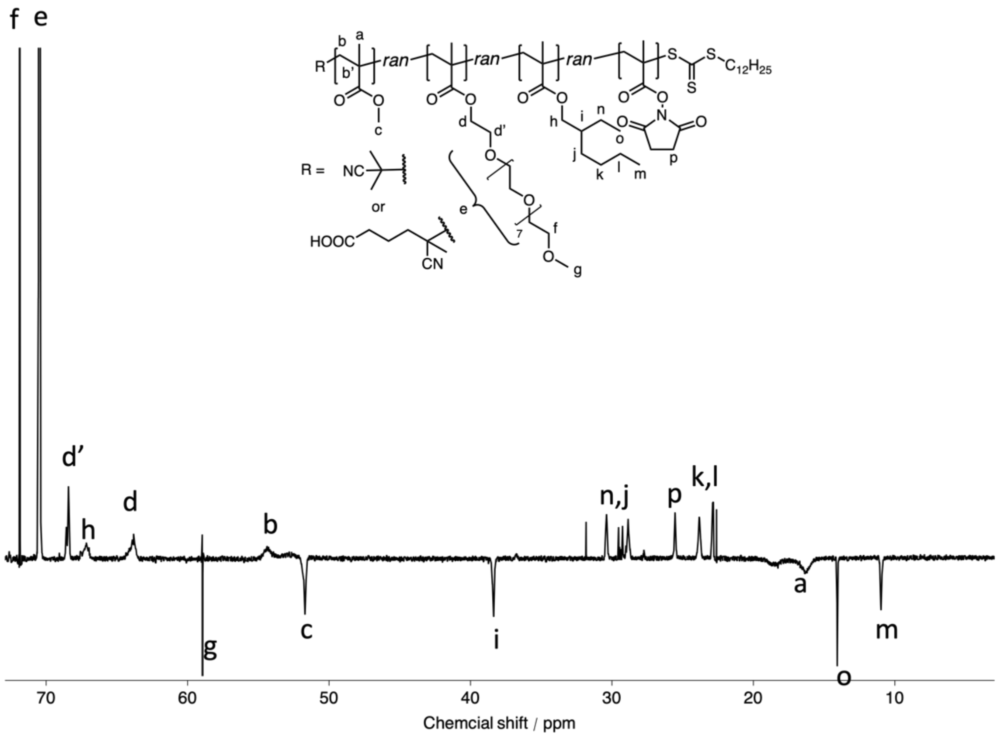

Figure S13. DEPT135 ^{13}C NMR spectrum of **RHP-3** (125 MHz, $CDCl_3$).

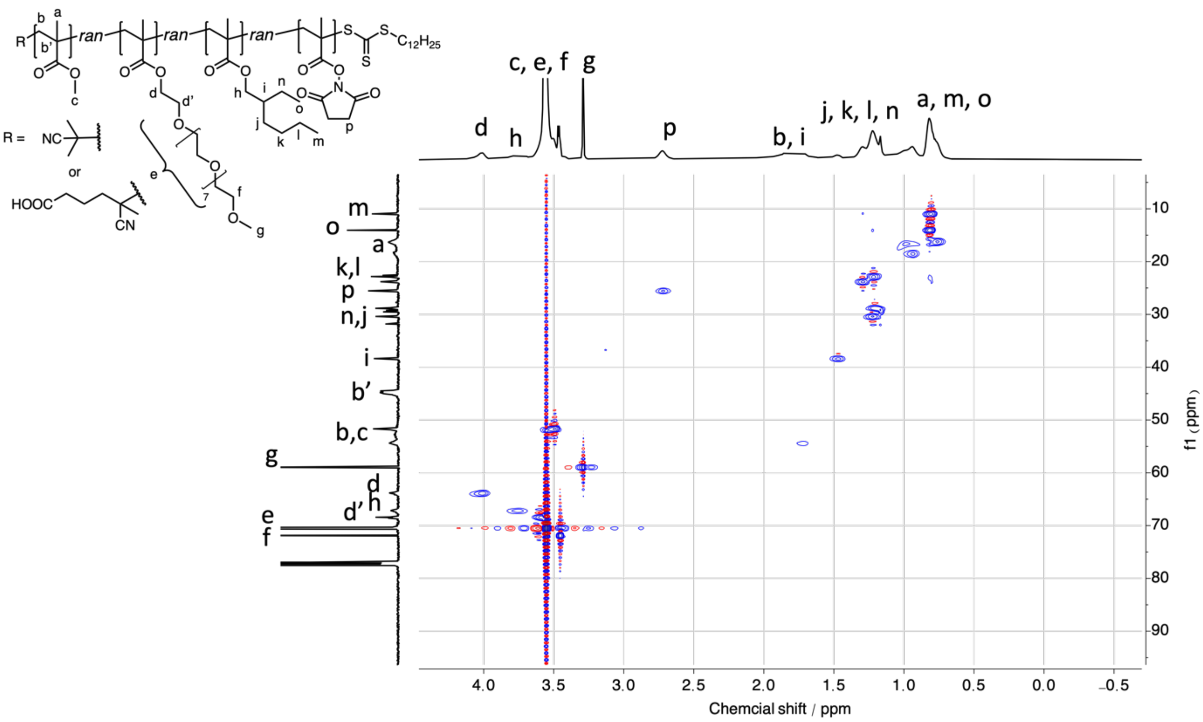

Figure S14. 2D ^{1}H-^{13}C HSQC NMR spectrum of **RHP-3** ($CDCl_3$).

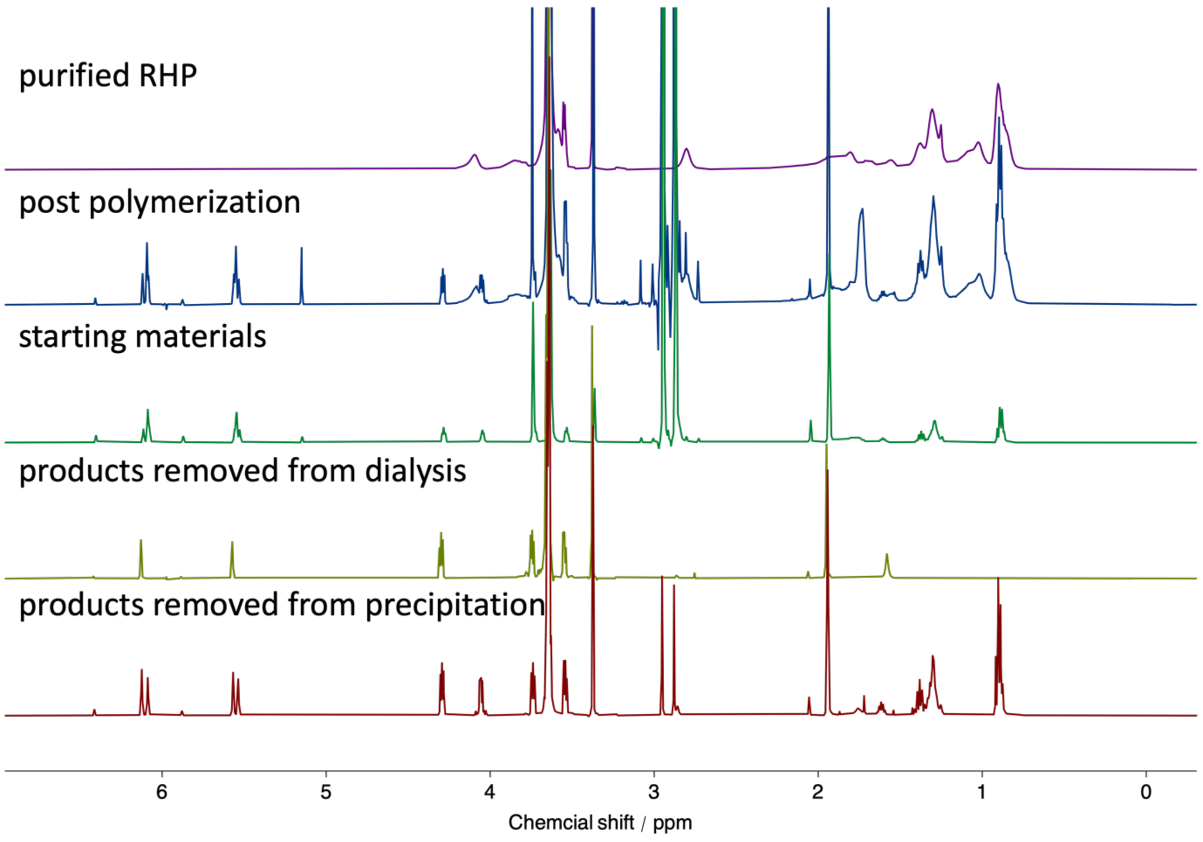

Figure S15. Comparison of ^{1}H NMR spectrum of **RHP-3**, crude products, starting materials, and products removed during purification (500 MHz, $CDCl_3$).

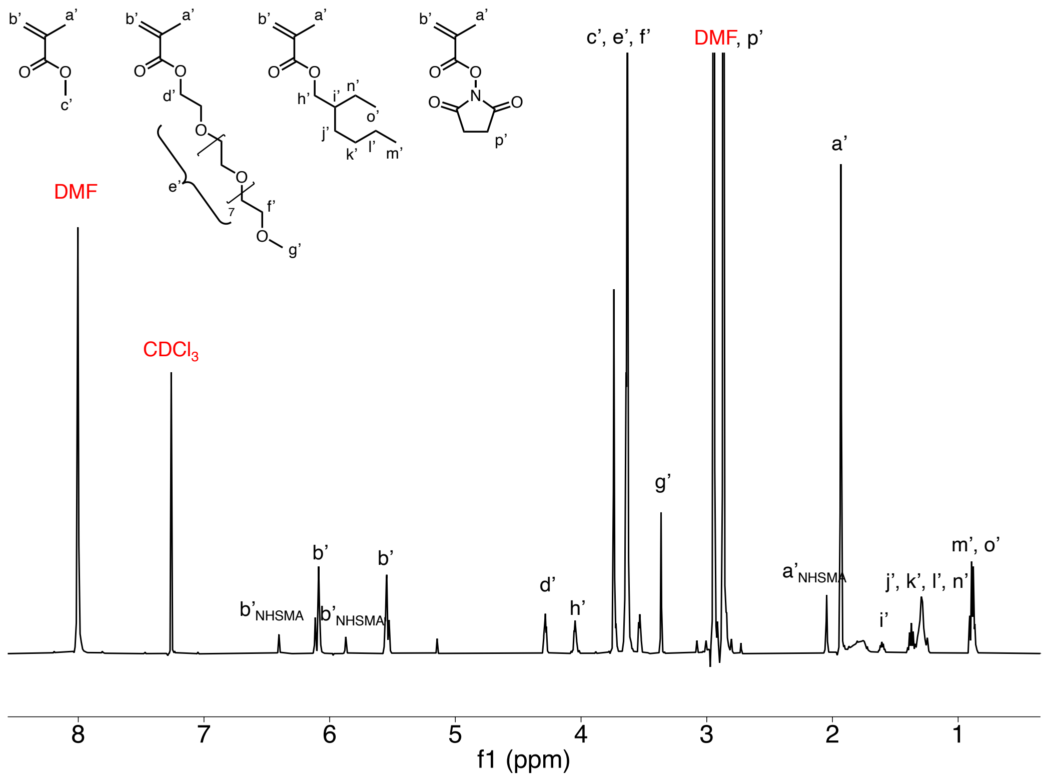

Figure S16. ^{1}H NMR spectrum of **RHP-3** pre-polymerization mixture (500 MHz, $CDCl_3$).

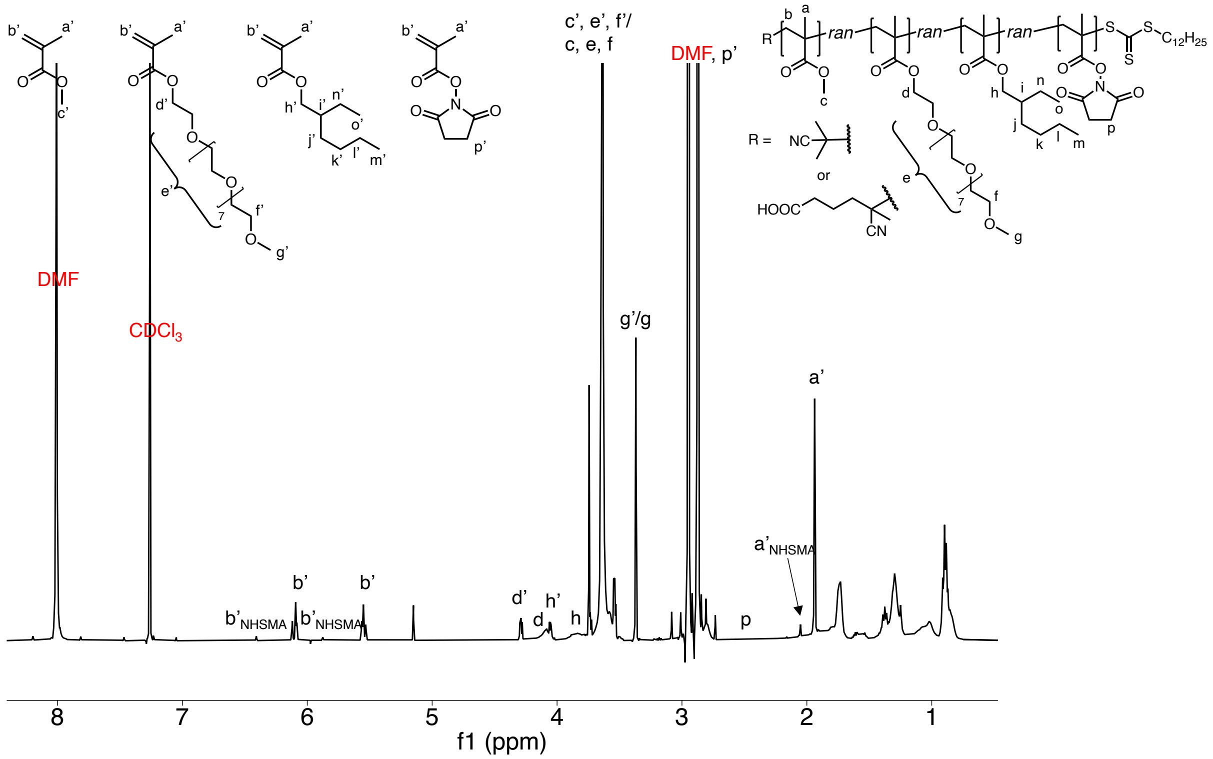

Figure S17. ¹H NMR spectrum of **RHP-3** post-polymerization mixture (500 MHz, $CDCl_3$).

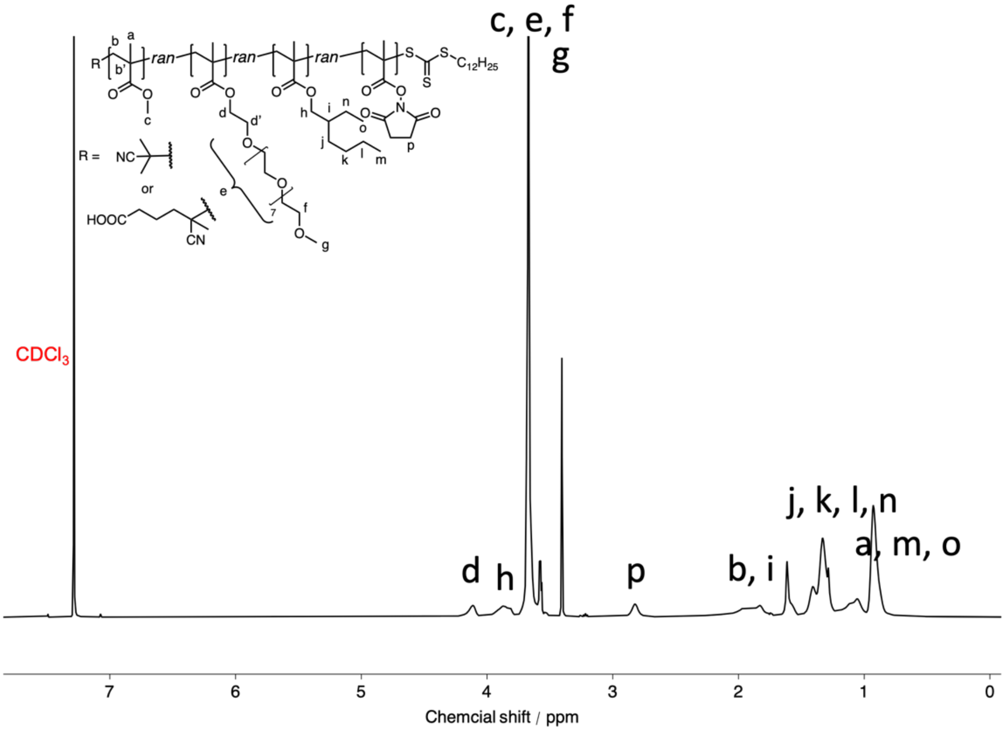

Figure S18. ^{1}H NMR spectrum of **RHP-4** (500 MHz, $CDCl_3$).

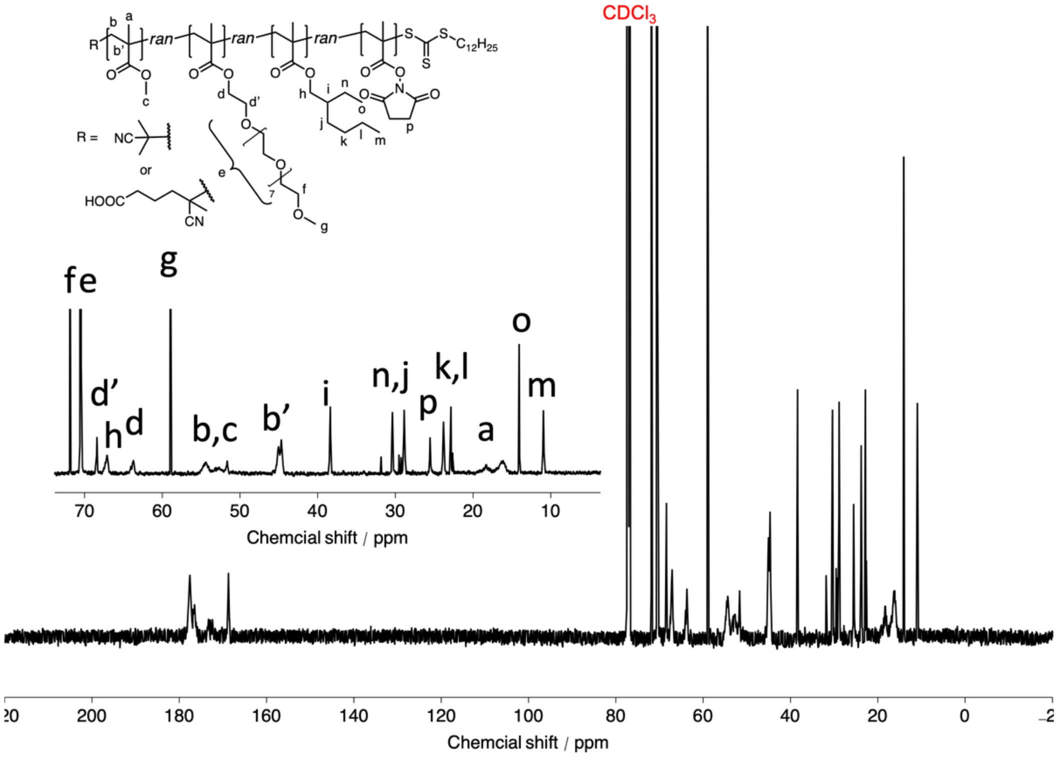

Figure S19. ^{13}C NMR spectrum of **RHP-4** (125 MHz, $CDCl_3$).

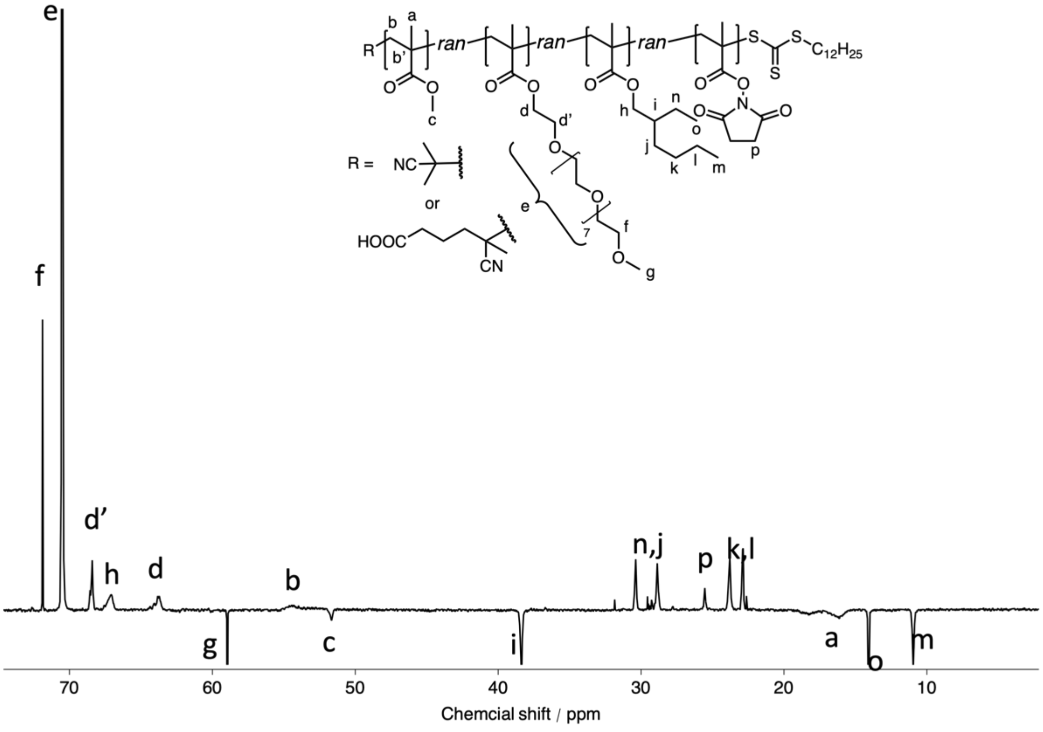

Figure S20. DEPT135 ^{13}C NMR spectrum of **RHP-4** (125 MHz, $CDCl_3$).

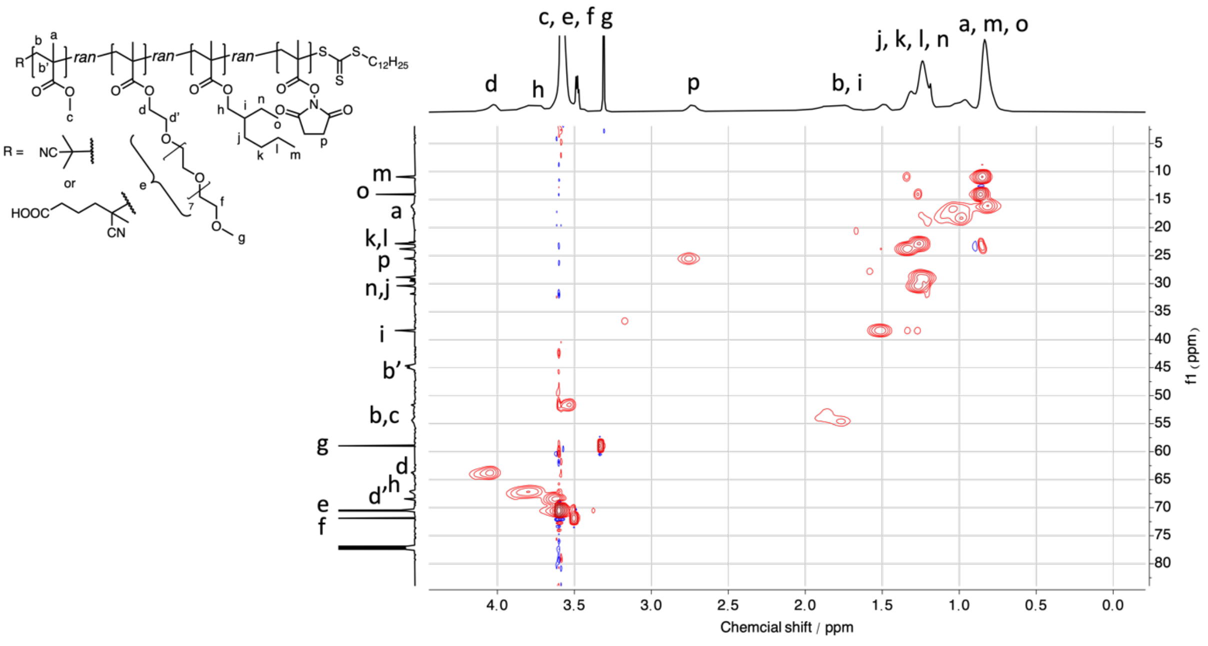

Figure S21. 2D ^{1}H-^{13}C HSQC NMR spectrum of **RHP-4** ($CDCl_3$).

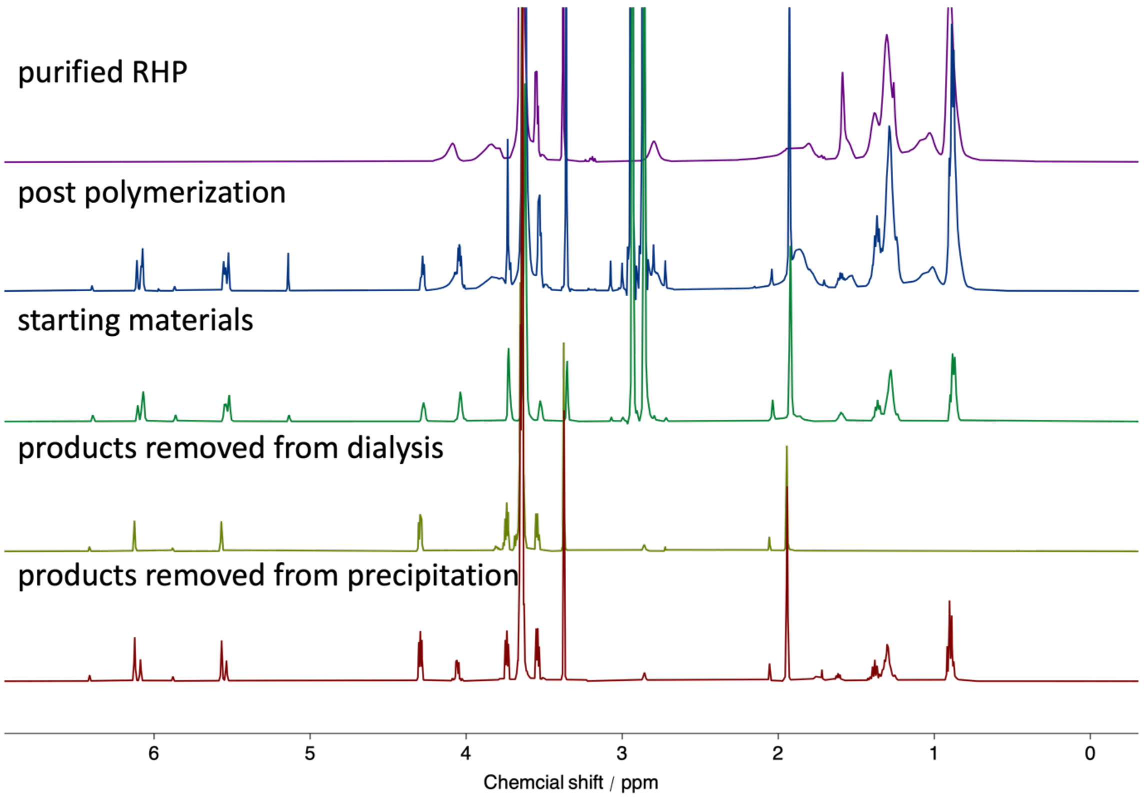

Figure S22. Comparison of ^{1}H NMR spectrum of **RHP-4**, crude products, starting materials, and products removed during purification (500 MHz, $CDCl_3$).

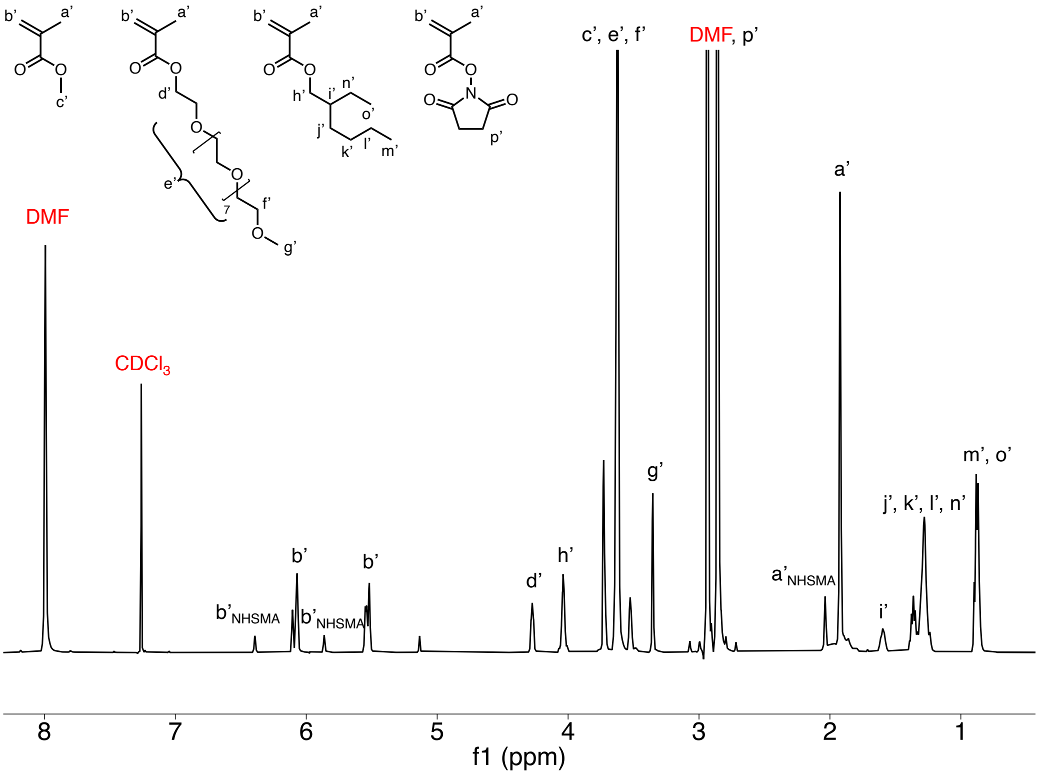

Figure S23. ^{1}H NMR spectrum of **RHP-4** pre-polymerization mixture (500 MHz, $CDCl_3$).

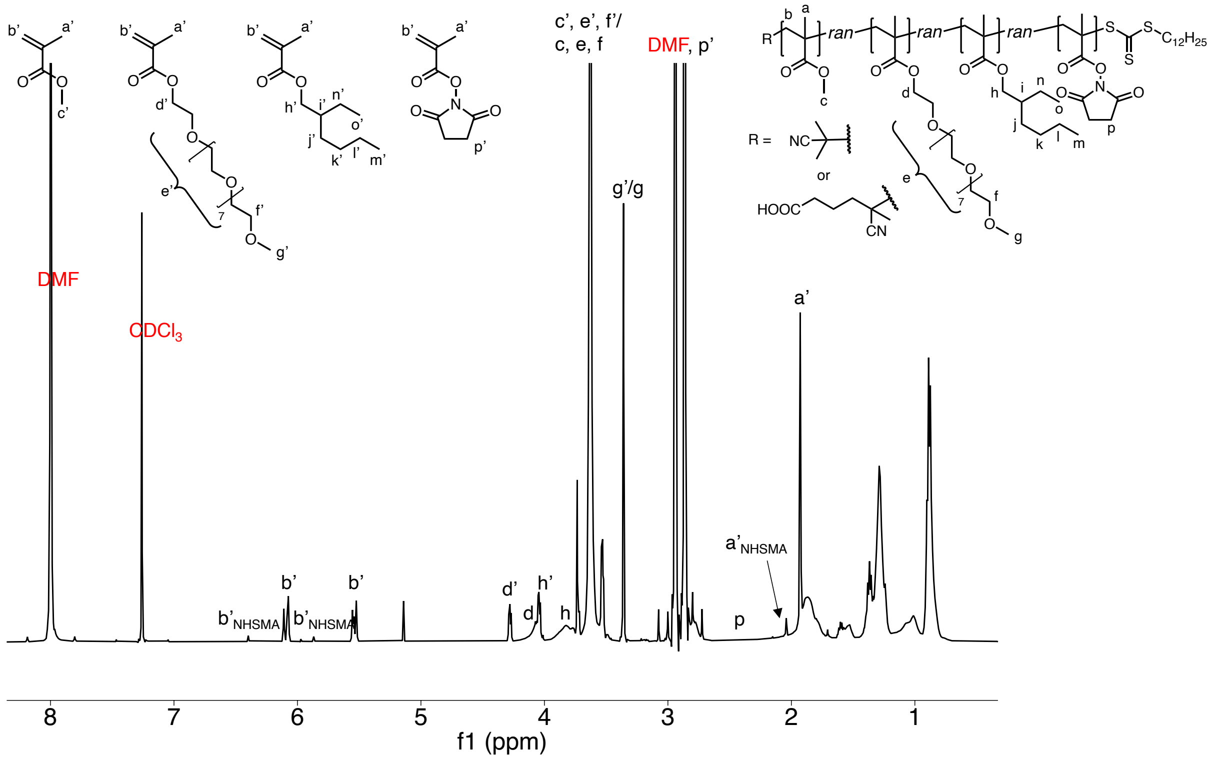

Figure S24. ^{1}H NMR spectrum of **RHP-4** post-polymerization mixture (500 MHz, $CDCl_3$).

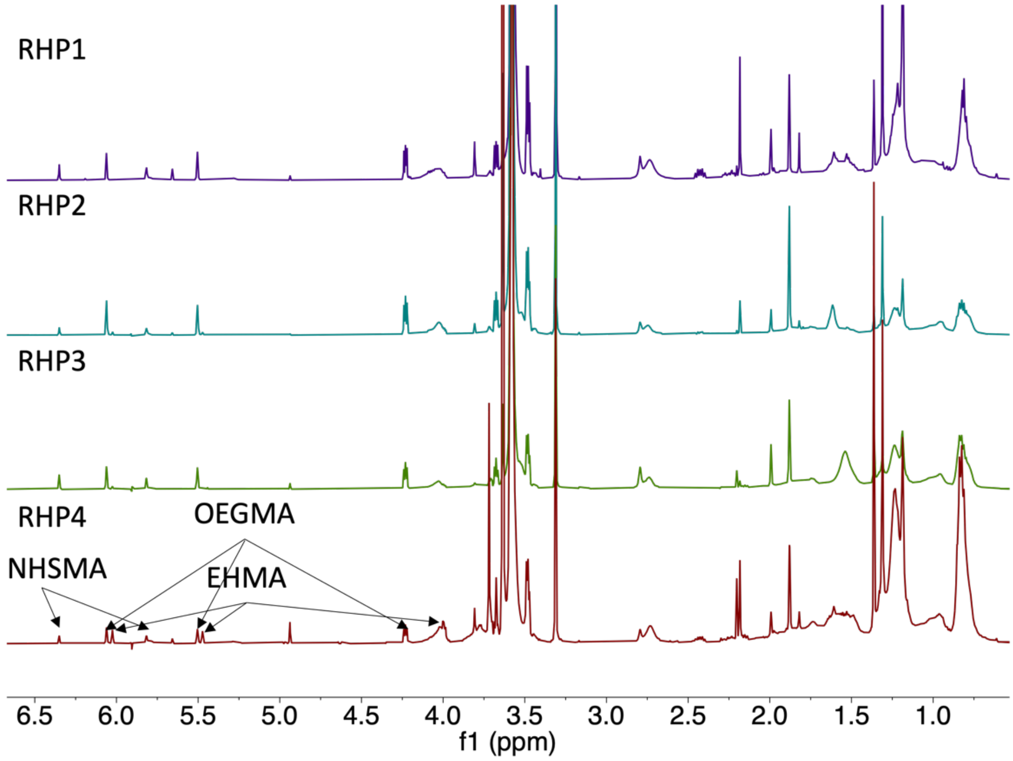

Figure S25. ^{1}H NMR spectrum (500 MHz, $CDCl_3$) of **RHP1-RHP4** after 3 hr depolymerization reaction (120 °C, dioxane). The majority of regenerated MMA was removed during concentration when preparing the NMR samples.

S.4 GPC and HPLC Results

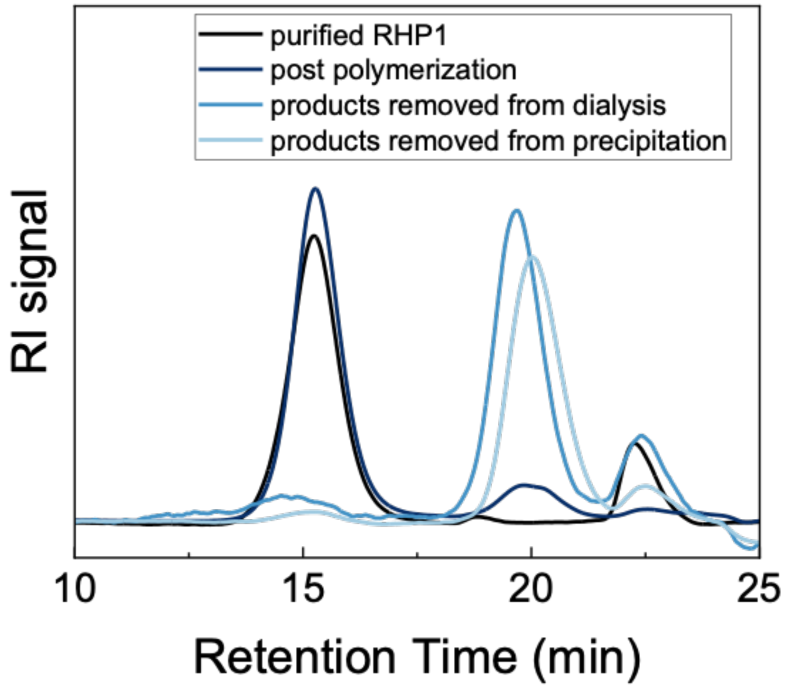

Figure S26. GPC traces of **RHP-1**, crude products, starting materials, and products removed during purification.

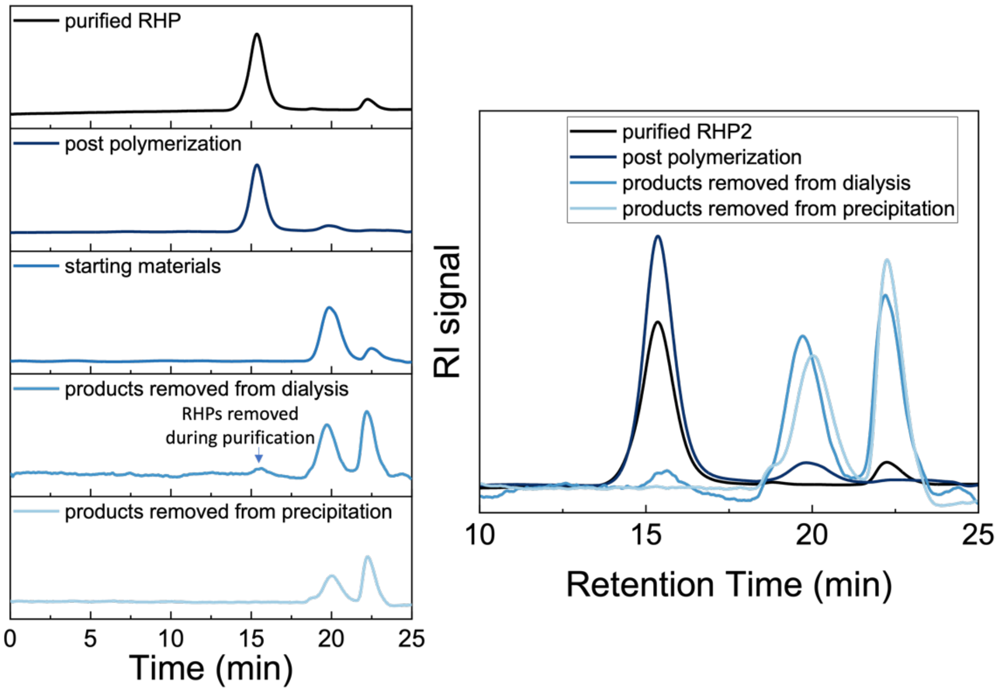

Figure S27. GPC traces of **RHP-2**, crude products, starting materials, and products removed during purification.

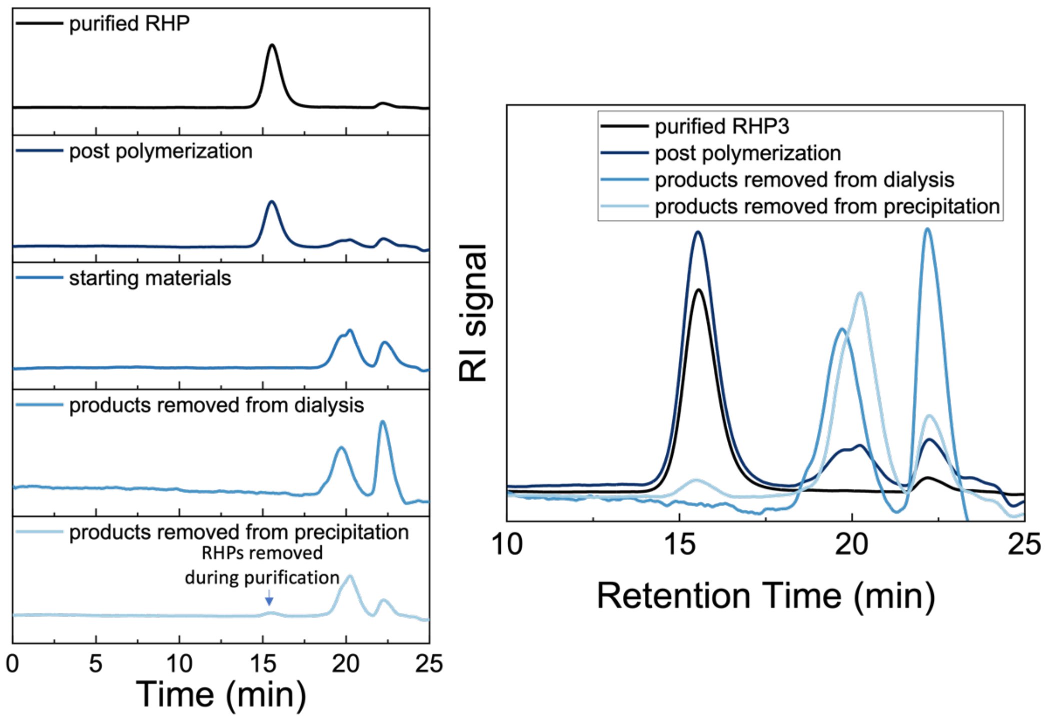

Figure S28. GPC traces of **RHP-3**, crude products, starting materials, and products removed during purification.

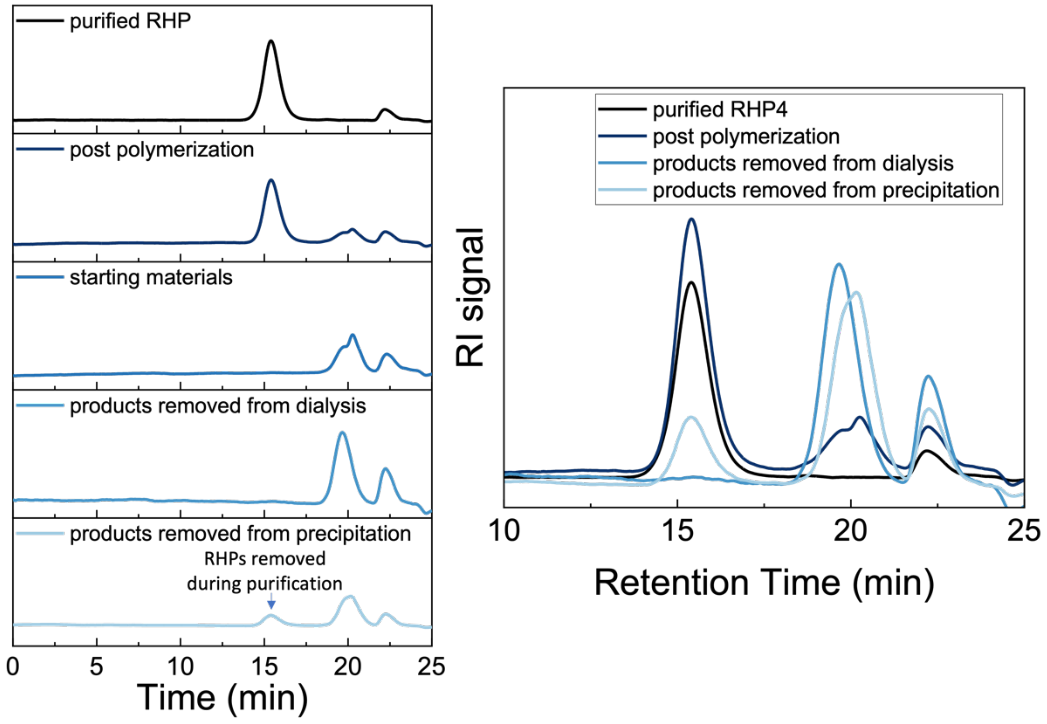

Figure S29. GPC traces of **RHP-4**, crude products, starting materials, and products removed during purification.

Table S5. Summary of representative GPC analysis (THF) on supernatant, dialysate, and purified RHPs. The ranges between 12.5 to 17.5 min were quantified using a PMMA calibration curve. The results of supernatant and dialysate reflects the RHPs subpopulations (>7 kDa based on PMMA standard) that were removed during post-polymerization purifications.

entry	Initial M_n (kDa)	*Đ*	Supernatant	*Đ*	Dialysate	*Đ*	Final M_n (kDa)	*Đ*
RHP1	20.1	1.34	26.5	1.19	36.0	1.36	23.7	1.40
RHP2	18.3	1.31	-	-	23.5	1.81	20.8	1.31
RHP3	17.2	1.34	19.5	1.23	-	-	13.3	1.36
RHP4	20.6	1.26	17.6	1.40	-	-	16.7	1.29

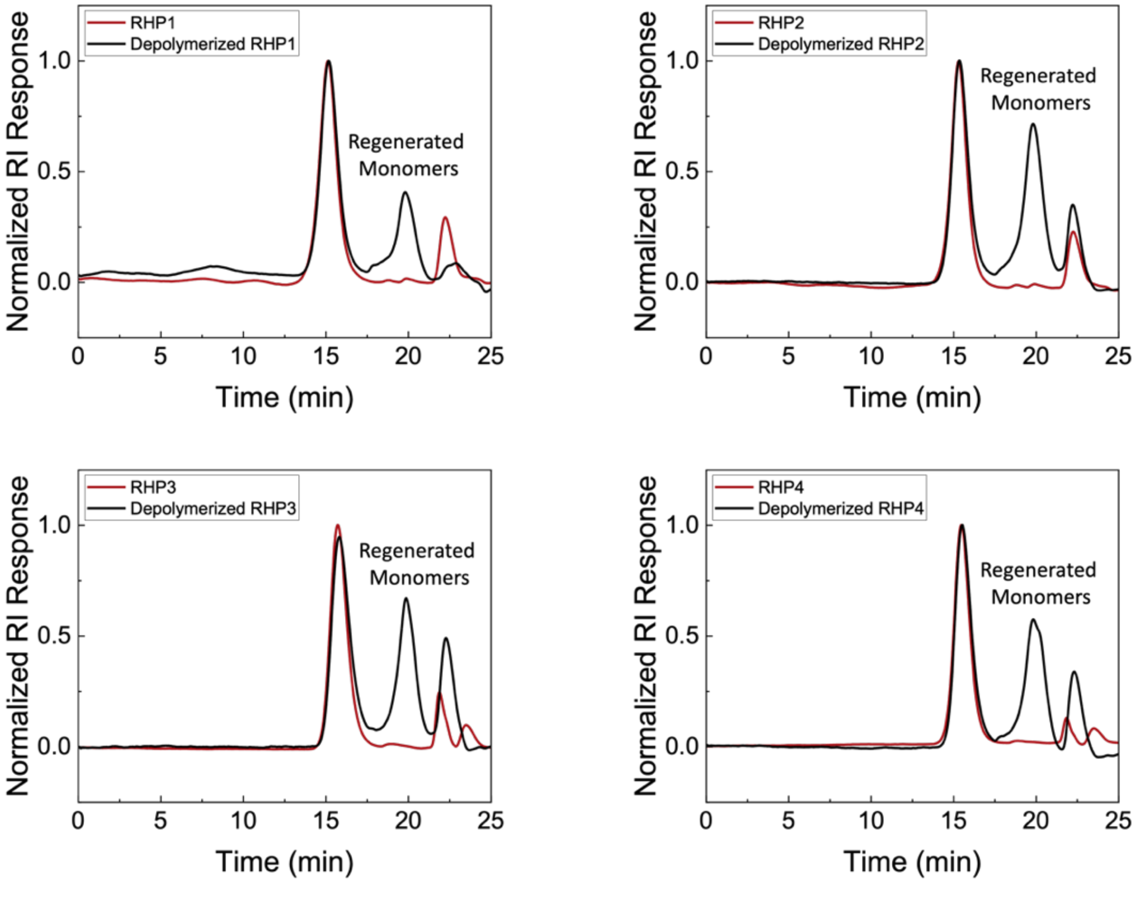

Figure S30. GPC traces (THF) of pristine and depolymerized RHPs after 3 hr depolymerization reaction.

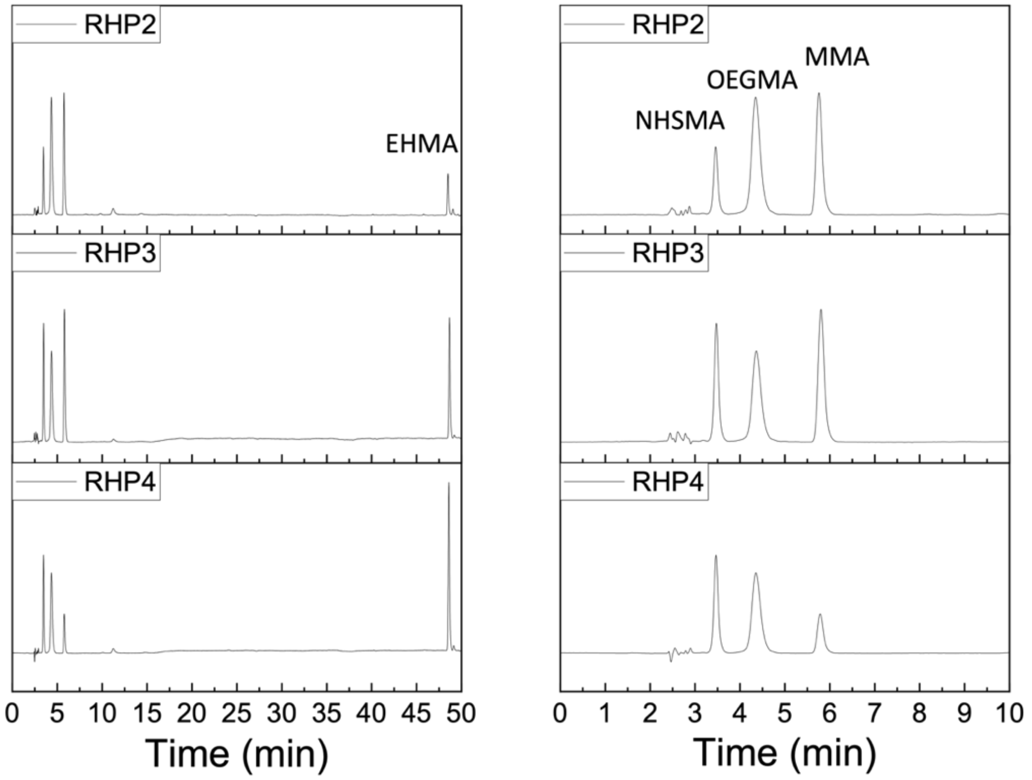

Figure S31. Representative HPLC traces of depolymerized **RHP-2**, **RHP-3**, and **RHP-4** (210 nm). Elution time: NHSMA (3.45 min), OEGMA (4.31 min), MMA (5.68 min) and EHMA (48.3 min). The peaks at 2.6 and 49 min are the residual solvent (dioxane) and the product from CTA thermal degradation, respectively.

Among the selected four monomers, the NHS ester of NHSMA is susceptible to hydrolysis during the dialysis. If the NHS ester of NHSMA was hydrolyzed during dialysis, we expect to observe methacrylic acid in the depolymerized RHPs mixtures. We therefore performed HPLC analysis on the depolymerization mixtures and methacrylic acid using the same conditions. The results show that there were no detectable amounts of methacrylic acid in the solution mixtures subsequent to depolymerization (**Figure S24**), supporting that the NHS ester remain largely intact during dialysis.

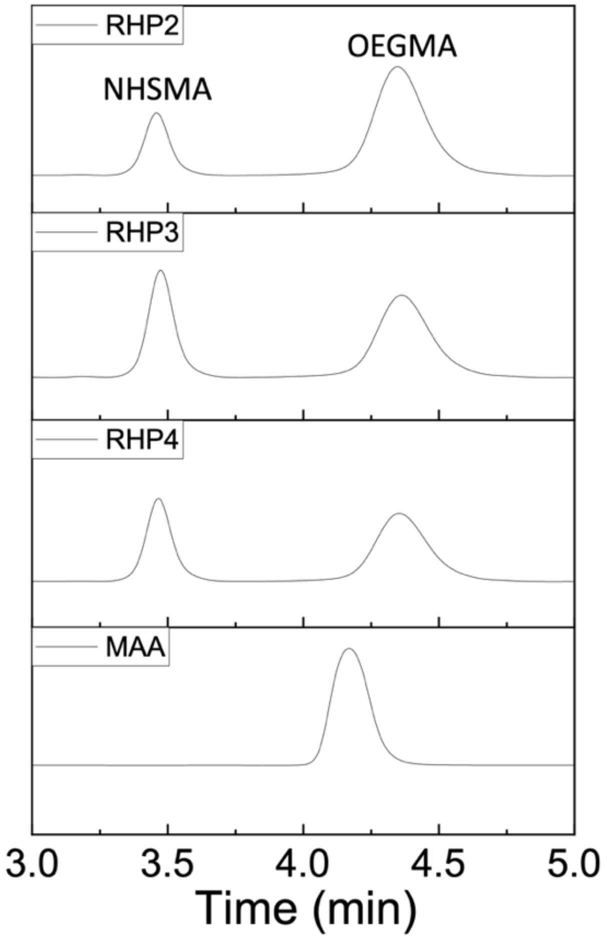

Figure S32. HPLC traces of depolymerized **RHP-2**, **RHP-3**, **RHP-4**, and methacrylic acid (MAA) (210 nm).

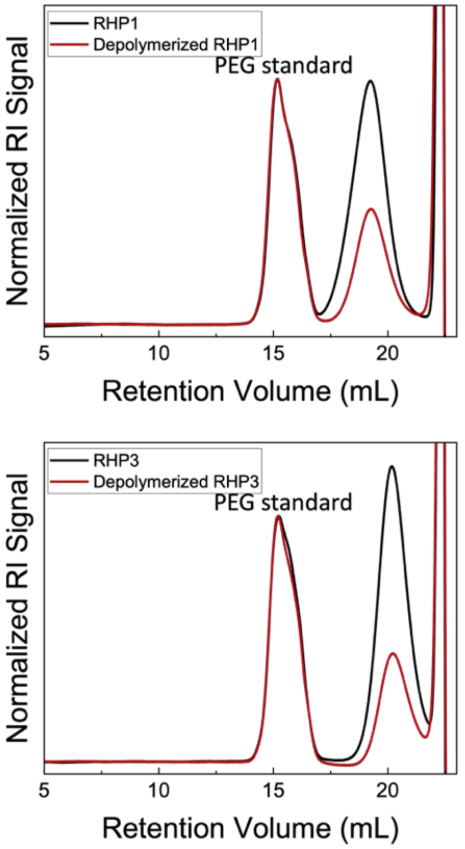

Figure S33. GPC traces (DMF) of pristine and depolymerized RHPs after 3 hr depolymerization reaction. The intensities of RHPs in pre- and post-depolymerization aliquots were normalized to an internal PEG standard (500 kDa) that was added at the beginning of the reaction. RHPs elute at 18.5-20 min and solvent (DMF) elutes at 24 min. The fraction areas of RHPs in pre- and post-depolymerization aliquots were calculated to determine the degree of depolymerization. The depolymerization conversions for **RHP1-4** after 3 hr were estimated as 57%, 39%, 60%, and 23%, respectively. In our model RHPs, a 5:1 CTA/initiator (AIBN) ratio was used in RHPs synthesis. Considering the half-life of AIBN at 80 °C is about 1.5 hr,[14,15] we therefore estimate that the dead chains account for approximately 23% of the whole chain ensemble of **RHP1-2** (synthesized at 80 °C in 2.5 hr), and 18% for **RHP3-4** (synthesized at 80 °C in 1.5 hr).

S.5 Determining Monomer Reactivity Ratios

Following the Jaacks method,[16,17] the monomer reactivity ratios were measured by conducting RAFT polymerization with a large excess of one monomer. For example, the feeding of monomer 1/monomer 2/monomer 3/ monomer 4 was 20/1/1/1. The equations below were used to determine the reactivity ratios.

$$r_{12} = Ln\frac{[M_1]_t}{[M_1]_0}/Ln\frac{[M_2]_t}{[M_2]_0};$$

$$r_{13} = Ln\frac{[M_1]_t}{[M_1]_0}/Ln\frac{[M_3]_t}{[M_3]_0};$$

$$r_{14} = Ln\frac{[M_1]_t}{[M_1]_0}/Ln\frac{[M_4]_t}{[M_4]_0};$$

In all cases, the ratio of total monomer/CTA (4-Cyano-4-[(dodecylsulfanylthiocarbonyl)sulfanyl]pentanoic acid)/initiator (AIBN) is 100/1/0.02. The reactions were run at 80 °C in DMF at the monomer concentration of 4 M. These experiments were conducted under the same conditions as that used in RHPs synthesis to take account of the influence of RAFT equilibrium on the propagation kinetics. Reactions with excess EHMA or NHSMA were run for 0.5 hr to avoid precipitation during polymerization. Reactions with excess OEGMA or MMA were run for 1-1.5 hr. At the given time, the reaction mixture was quenched in liquid nitrogen and aliquot was taken for NMR analysis to determine the consumption of each monomer by comparing with pre-polymerization aliquot. The reactivity ratio $r_{OEGMA/EHMA}$ and $r_{OEGMA/MMA}$ were determined separately by two-monomer copolymerization experiments to minimize analysis error due to peak overlap. Each experiment was repeated at least three times and a total of 18 RAFT polymerization experiments were performed to determine the average reactivity ratio.

Table S6. Summary of monomer conversion in the reactivity ratio determination triplicate experiments.

**MMA excess**	**MMA**	**EHMA**	**OEGMA**	**EHMA**
1	66%	70%	64%	43%
2	63%	73%	61%	39%
3	63%	70%	61%	37%
**EHMA excess**	**MMA**	**EHMA**	**OEGMA**	**EHMA**
1	65%	39%	31%	56%
2	82%	40%	31%	56%
3	72%	42%	39%	59%
**OEGMA excess**	**MMA**	**EHMA**	**OEGMA**	**EHMA**
1	NA	NA	66%	79%
2	NA	NA	69%	86%
3	NA	NA	71%	83%
**NHSMA excess**	**MMA**	**EHMA**	**OEGMA**	**EHMA**
1	81%	56%	62%	74%
2	88%	47%	56%	73%
3	87%	46%	53%	73%

**OEGMA excess (two-monomer copolymerization)**	**MMA**	**OEGMA**
1	38%	27%
2	55%	32%
3	37%	36%
**OEGMA excess (two-monomer copolymerization)**	**EHMA**	**OEGMA**
1	59%	33%
2	60%	35%
3	75%	37%

Among the 12 reactivity ratios measured in this work, the reactivity ratios of $r_{NHSMA/OEGMA}$ (0.9), $r_{OEGMA/NHSMA}$ (0.7), $r_{NHSMA/MMA}$ (0.7), and $r_{MMA/NHSMA}$ (1.3) in free radical polymerization have been reported previously.[18,19] Compared to prior work, we observed different values of $r_{NHSMA/OEGMA}$ (1.6) and $r_{MMA/NHSMA}$ (0.5) in the RAFT polymerization. We attributed the differences to the variations in polymerization conditions such as solvent, temperature, and concentration. Moreover, prior work has indicated that the different time scales of monomer addition in free and controlled radical polymerization (ATRP) affect the reactivity ratios.[20] We therefore conjecture that the RAFT equilibrium can also influence the reactivity ratios for specific monomer pairs.

S.6 Summary of Composition Ratios

Table S7. Summary of composition ratio of raw RHPs calculated from pre/post-polymerization aliquot NMR. (n = 3)[21,22]

# raw RHPs	MMA (%)	EHMA (%)	OEGMA (%)	NHSMA (%)
RHP1	3.3 ± 0.4	20.2 ± 2.4	48.1 ± 2.5	28.3 ± 1.9
RHP2	30.0 ± 2.0	11.8 ± 2.1	46.1 ± 1.6	12.2 ± 0.8
RHP3	41.1 ± 5.3	22.4 ± 2.5	22.0 ± 1.6	13.9 ± 1.2
RHP4	20.1 ± 2.5	41.6 ± 1.3	27.2 ± 1.7	11.1 ± 1.3

Table S8. Summary of composition ratio of raw RHPs simulated using *RHPapp*.

# simulated RHPs	MMA (%)	EHMA (%)	OEGMA (%)	NHSMA (%)
RHP1	4.3	22.3	45.7	27.7
RHP2	32.3	12.6	43.3	11.8
RHP3	45.3	20.8	20.6	13.3
RHP4	19.2	45.8	24.1	10.9

Table S9. Summary of composition ratio of purified RHPs determined by quantitative ^{13}C NMR. (n = 3)

# purified RHPs	MMA (%)	EHMA (%)	OEGMA (%)	NHSMA (%)
RHP1	11.0 ± 1.9	17.2 ± 1.2	47.5 ± 1.2	24.3 ± 1.6
RHP2	31.5 ± 1.0	9.6 ± 6.4	48.1 ± 0.9	11.2 ± 4.7
RHP3	37.6 ± 4.8	23.3 ± 1.3	24.2 ± 1.9	15.0 ± 1.7
RHP4	17.2 ± 0.7	45.7 ± 5.9	23.6 ± 1.0	13.4 ± 7.4

Table S10. Summary of composition ratio of RHPs living chains calculated from RHPs depolymerization experiments. (n = 9)

# living chains in RHPs	MMA (%)	EHMA (%)	OEGMA (%)	NHSMA (%)
RHP1	4.1 ± 2.8	19.8 ± 3.3	45.5 ± 7.4	30.6 ± 11.0
RHP2	24.2 ± 6.1	14.8 ± 5.0	49.8 ± 4.9	11.2 ± 3.0
RHP3	27.4 ± 5.4	24.7 ± 2.9	28.3 ± 1.7	19.6 ± 1.8
RHP4	7.6 ± 1.5	43.0 ± 4.2	32.3 ± 5.1	17.1 ± 1.6

S.7 Additional Analysis

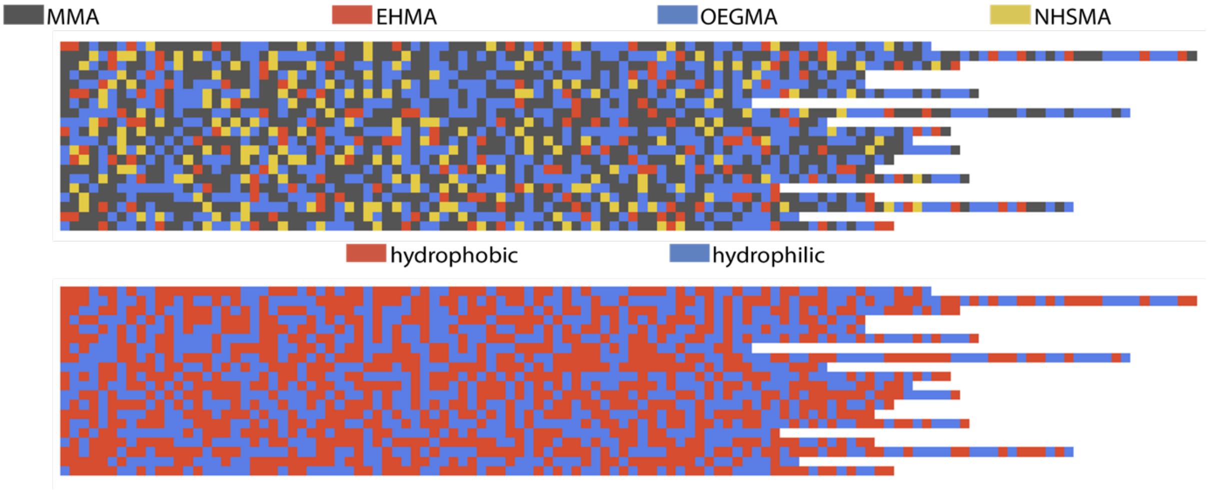

Figure S34. Random sequences sampled from simulated **RHP2** using measured reactivity ratios and targeted monomer conversion. The RHP sequences shown at the bottom were binarized into hydrophobic and hydrophilic units to display the distributions of hydrophobic/hydrophilic segments.

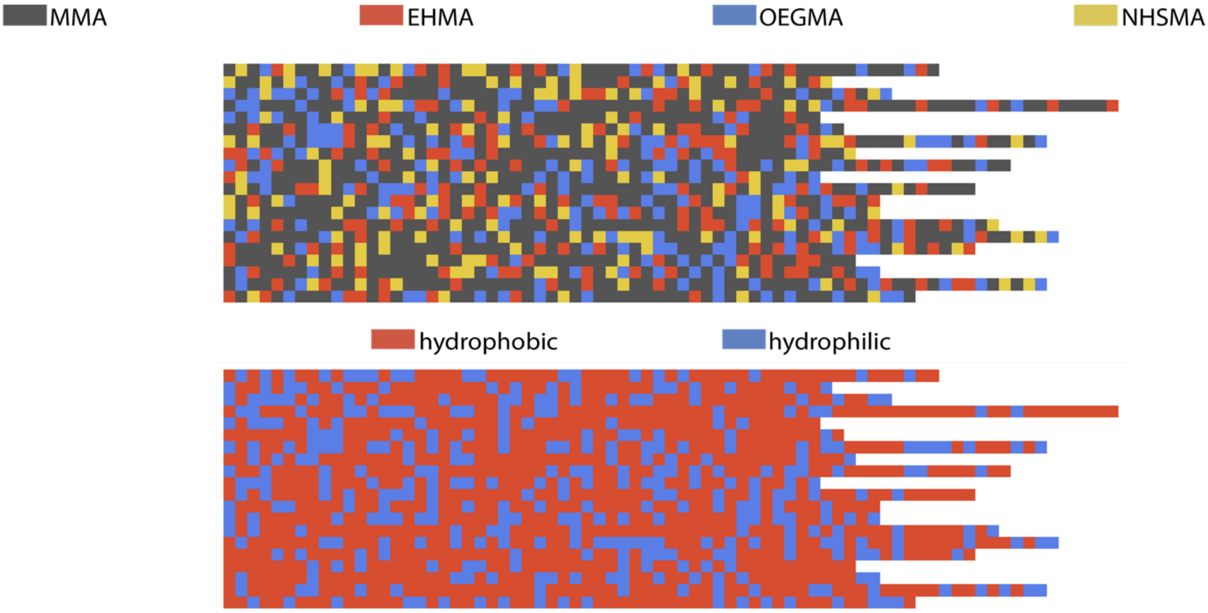

Figure S35. Random sequences sampled from simulated **RHP3** using measured reactivity ratios and targeted monomer conversion. The RHP sequences shown at the bottom were binarized into hydrophobic and hydrophilic units to display the distributions of hydrophobic/hydrophilic segments.

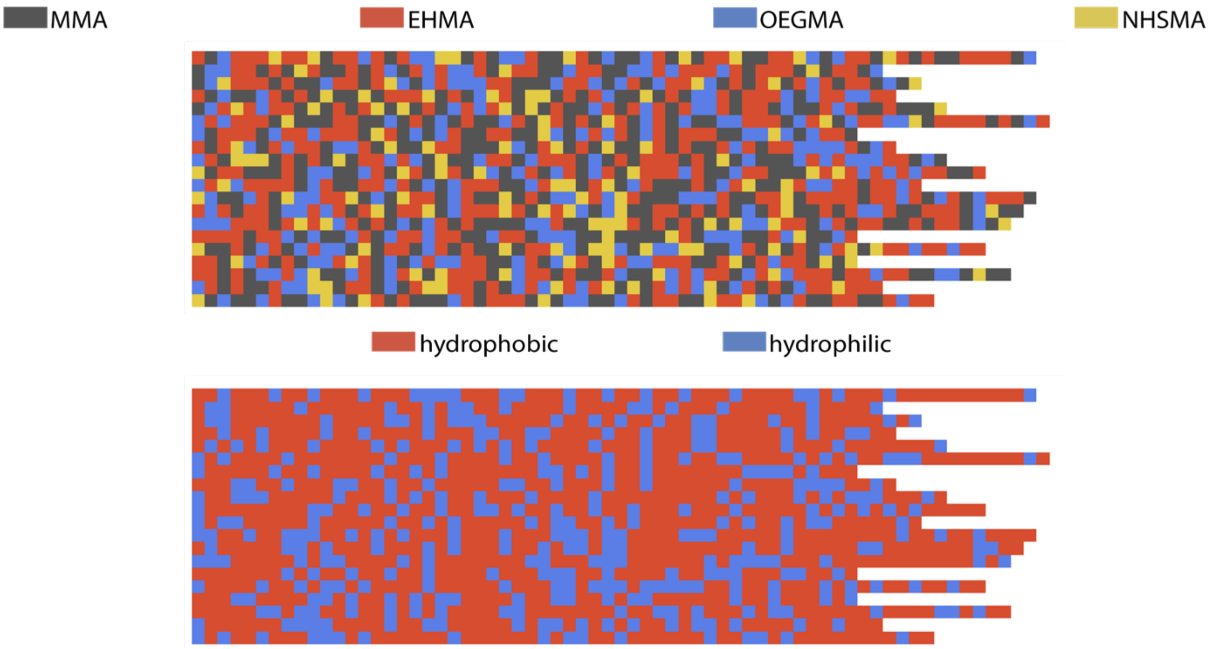

Figure S36. Random sequences sampled from simulated **RHP4** using measured reactivity ratios and targeted monomer conversion. The RHP sequences shown at the bottom were binarized into hydrophobic and hydrophilic units to display the distributions of hydrophobic/hydrophilic segments.

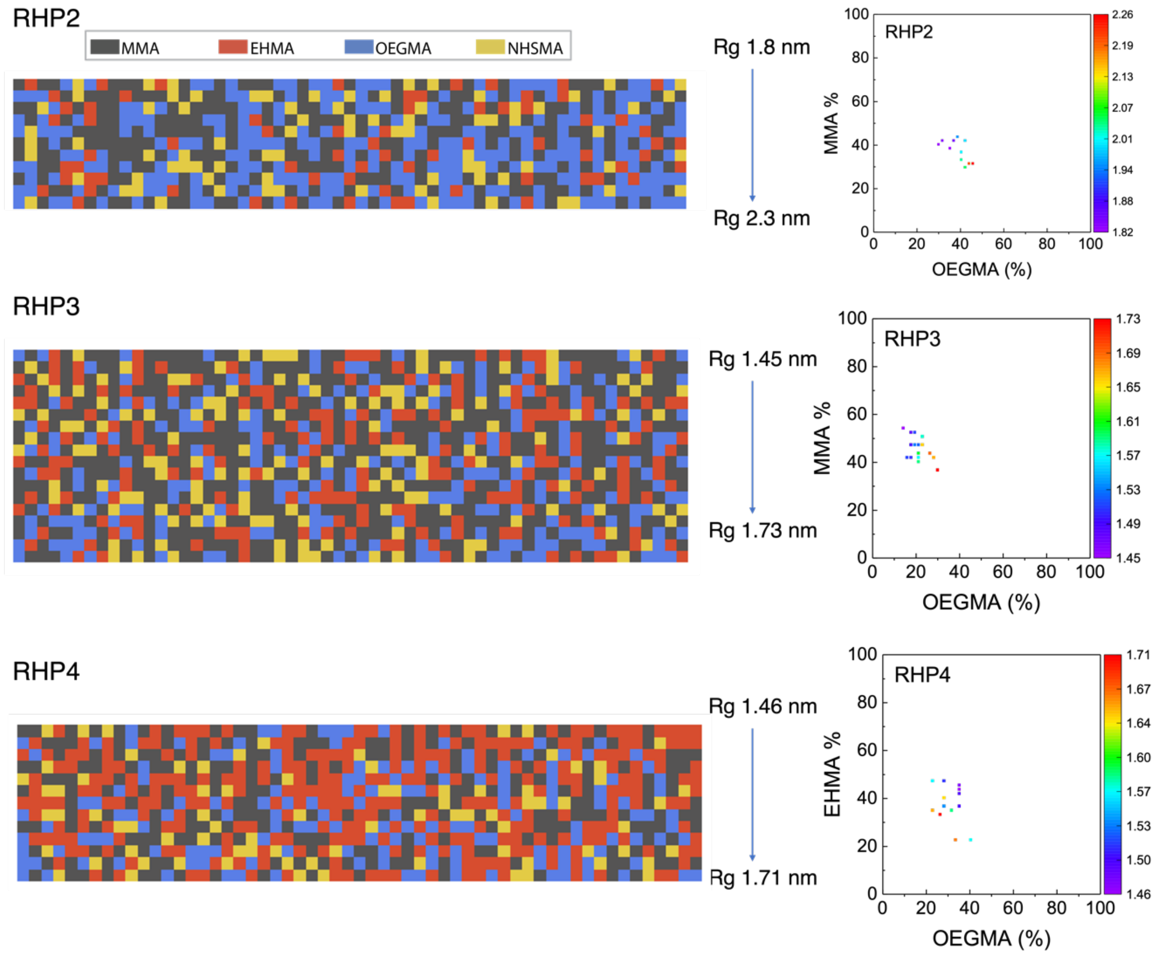

Figure S37. Sequences with identical chain length (N = 57) sampled from the simulated **RHP2-4** ensembles. The sequences presented are sorted in descending order of R_g (water).

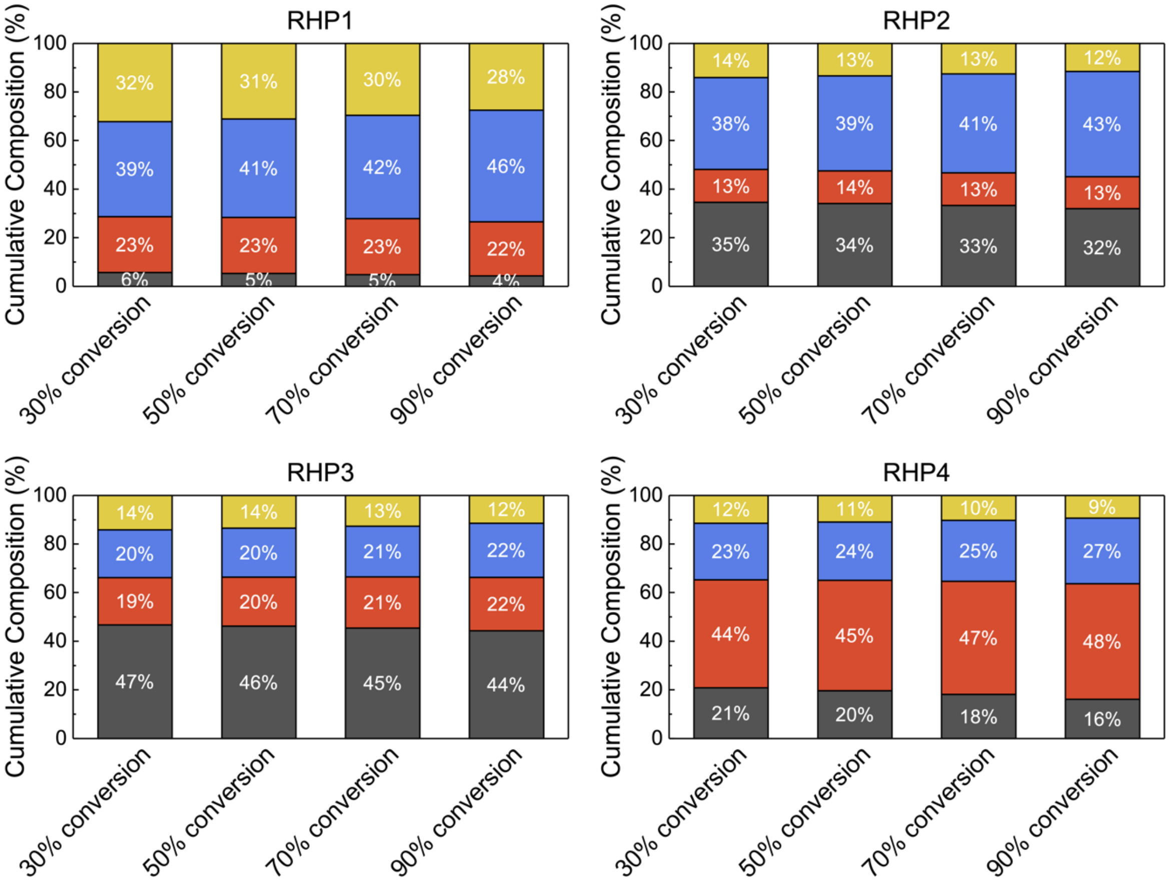

Figure S38. Simulated composition drift during RAFT polymerization at different monomer conversions. Simulation results reflect the average composition of 50,000 simulated individual chains.

S.8 References

(1) Gimenes De Souza, C.; Torres De Araújo, M.; Cavalcante Dos Santos, R.; França De Andrade, D.; Vasconcello Da Silva, B.; Davila, L. A. Analysis and Quantitation of Fatty Acid Methyl Esters in Biodiesel by High-Performance Liquid Chromatography. *Energy and Fuels* **2018**, *32* (11), 11547–11554.

(2) Jayapurna, I.; Ruan, Z.; Eres, M.; Jalagam, P.; Jenkins, S.; Xu, T. Sequence Design of Random Heteropolymers as Protein Mimics. *Biomacromolecules* **2023**, *24* (2), 652–660.

(3) Davies, J. T. *A QUANTITATIVE KINETIC THEORY OF EMULSION TYPE. I. PHYSICAL CHEMISTRY OF THE EMULSIFYING AGENT.*

(4) Jiang, T.; Hall, A.; Eres, M.; Hemmatian, Z.; Qiao, B.; Zhou, Y.; Ruan, Z.; Couse, A. D.; Heller, W. T.; Huang, H.; de la Cruz, M. O.; Rolandi, M.; Xu, T. Single-Chain Heteropolymers Transport Protons Selectively and Rapidly. *Nature* **2020**, *577* (7789), 216–220.

(5) Guo, X.; Rong, Z.; Ying, X. Calculation of Hydrophile-Lipophile Balance for Polyethoxylated Surfactants by Group Contribution Method. *J Colloid Interface Sci* **2006**, *298* (1), 441–450.

(6) Fredrickson, G. *The Equilibrium Theory of Inhomogeneous Polymers*; International Series of Monographs on Physics; OUP Oxford: Oxford, 2006; Vol. 134.

(7) Wang, R.; Wang, Z.-G. Theory of Polymers in Poor Solvent: Phase Equilibrium and Nucleation Behavior. *Macromolecules* **2012**, *45* (15), 6266–6271.

(8) Wang, R.; Wang, Z.-G. Theory of Polymer Chains in Poor Solvent: Single-Chain Structure, Solution Thermodynamics, and Θ Point. *Macromolecules* **2014**, *47* (12), 4094–4102.

(9) Mark, J. E. *Physical Properties of Polymers Handbook*, 2. Aufl.; Springer-Verlag: New York, NY, 2007.

(10) Lindvig, T.; Michelsen, M. L.; Kontogeorgis, G. M. A Flory–Huggins Model Based on the Hansen Solubility Parameters. *Fluid Phase Equilib* **2002**, *203* (1), 247–260.

(11) Hansen, C. M. *Hansen Solubility Parameters: A User's Handbook, Second Edition*, 2nd ed.; CRC Press: Milton, 2007.

(12) Eliassi, A.; Modarress, H.; Mansoori, G. A. Measurement of Activity of Water in Aqueous Poly(Ethylene Glycol) Solutions (Effect of Excess Volume on the Flory−Huggins χ-Parameter). *J Chem Eng Data* **1999**, *44* (1), 52–55.

(13) Stribeck, N.; Smarsly, B. *Scattering Methods and the Properties of Polymer Materials*, 1st ed.; Progress in Colloid and Polymer Science; Springer Berlin Heidelberg: Berlin, Heidelberg, 2005; Vol. 130.

(14) Kim, K.; Ahn, J.; Park, M.; Lee, H.; Kim, Y. J.; Chang, T.; Jeon, H. B.; Paik, H. J. Molecular-Weight Distribution of Living Chains in Polystyrene Prepared by Reversible Addition-Fragmentation Chain-Transfer Polymerization. *Macromolecules* **2019**, *52* (19), 7448–7455.

(15) Van Hook, J. P.; Tobolsky, A. V. The Thermal Decomposition of 2,2'-Azo-Bis-Isobutyronitrile. *J Am Chem Soc* **1958**, *80* (4), 779–782.

(16) Jaacr~, V. A Novel Method of Determination of Reactivity Ratios in Binary and Ternary Copolymerizations **I. *Die Makromolekulare Chemie* **1972**, *161*, 161–172.

(17) Roos, S. G.; Müller, A. H. E.; Matyjaszewski, K. Copolymerization of Rc-Butyl Acrylate with Methyl Methacrylate and Pmma Macromonomers: Comparison of Reactivity Ratios in Conventional and Atom Transfer Radical Copolymerization. *Macromolecules* **1999**, *32* (25), 8331–8335.

(18) Alb, A. M.; Enohnyaket, P.; Drenski, M. F.; Shunmugam, R.; Tew, G. N.; Reed, W. F. Quantitative Contrasts in the Copolymerization of Acrylate- and Methacrylate-Based Comonomers. *Macromolecules* **2006**, *39* (24), 8283–8292.
(19) Viswanath, A.; Shen, Y.; Green, A. N.; Tan, R.; Greytak, A. B.; Benicewicz, B. C. Copolymerization and Synthesis of Multiply Binding Histamine Ligands for the Robust Functionalization of Quantum Dots. *Macromolecules* **2014**, *47* (23), 8137–8144.
(20) Roos, S. G.; Müller, A. H. E.; Matyjaszewski, K. Copolymerization of N-Butyl Acrylate with Methyl Methacrylate and PMMA Macromonomers: Comparison of Reactivity Ratios in Conventional and Atom Transfer Radical Copolymerization. *Macromolecules* **1999**, *32* (25), 8331–8335.
(21) Kafouris, D.; Kossivas, F.; Constantinides, C.; Nguyen, N. Q.; Wesdemiotis, C.; Patrickios, C. S. Biosourced Amphiphilic Degradable Elastomers of Poly(Glycerol Sebacate): Synthesis and Network and Oligomer Characterization. *Macromolecules* **2013**, *46* (3), 622–630.
(22) Holmberg, A. L.; Karavolias, M. G.; Epps, T. H. RAFT Polymerization and Associated Reactivity Ratios of Methacrylate-Functionalized Mixed Bio-Oil Constituents. *Polym Chem* **2015**, *6* (31), 5728–5739.
